# Efficient catalytic alkyne metathesis with a fluoroalkoxy-supported ditungsten(III) complex

**DOI:** 10.3762/bjoc.14.220

**Published:** 2018-09-18

**Authors:** Henrike Ehrhorn, Janin Schlösser, Dirk Bockfeld, Matthias Tamm

**Affiliations:** 1 Institut für Anorganische und Analytische Chemie, Technische Universität Braunschweig, Hagenring 30, 38106 Braunschweig, Germany

**Keywords:** alkylidyne complexes, alkyne metathesis, catalysis, terminal alkynes, tungsten

## Abstract

The molybdenum and tungsten complexes M_2_(OR)_6_ (**Mo2F6**, M = Mo, R = C(CF_3_)_2_Me; **W2F3**, M = W, R = OC(CF_3_)Me_2_) were synthesized as bimetallic congeners of the highly active alkyne metathesis catalysts [MesC≡M{OC(CF_3_)*_n_*Me_3−_*_n_*}] (**MoF6**, M = Mo, *n* = 2; **WF3**, M = W, *n* = 1; Mes = 2,4,6-trimethylphenyl). The corresponding benzylidyne complex [PhC≡W{OC(CF_3_)Me_2_}] (**W^Ph^F3**) was prepared by cleaving the W≡W bond in **W2F3** with 1-phenyl-1-propyne. The catalytic alkyne metathesis activity of these metal complexes was determined in the self-metathesis, ring-closing alkyne metathesis and cross-metathesis of internal and terminal alkynes, revealing an almost equally high metathesis activity for the bimetallic tungsten complex **W2F3** and the alkylidyne complex **W^Ph^F3**. In contrast, **Mo2F6** displayed no significant activity in alkyne metathesis.

## Introduction

**Figure 1:**
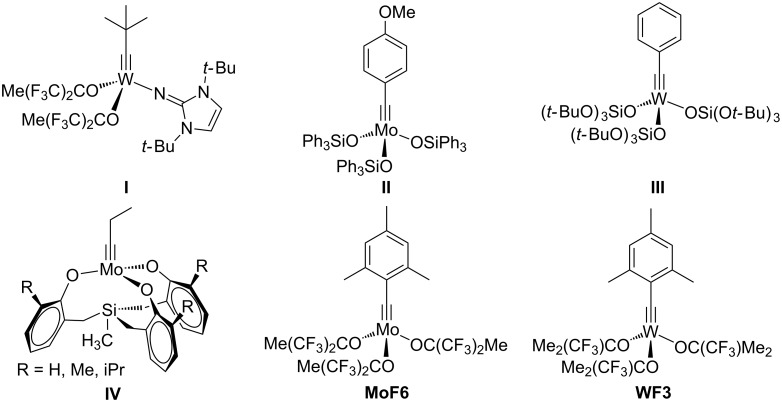
Selected homogeneous catalysts for alkyne metathesis.

While the field of olefin metathesis has seen significant progress in the past decades [1–5], the synthetic potential of alkyne metathesis has been growing only recently [6–11]. Alkyne metathesis represents a transition-metal-catalyzed transformation in which carbon–carbon triple bonds are cleaved and formed under mild conditions via metallacyclobutadiene (MCBD) intermediates [12]. Ongoing progress in the development of highly active homogeneous alkyne metathesis catalysts (Figure 1) has increased the value of this method in natural product and materials chemistry.

The contributions from our group to the development of alkyne metathesis catalysts were initially based on a design strategy inspired by the structure of highly active olefin metathesis catalysts, the Schrock-type molybdenum and tungsten alkylidene complexes [13–15]. Imidazolin-2-iminato ligands were used to modify Schrock-type alkylidyne complexes, resulting in new active alkyne metathesis catalysts such as **I** (Figure 1) [16–20]. Initially, the neopentylidyne tungsten complex was synthesized via the conventional “high-oxidation-state route” developed by R. R. Schrock [16–17,21–22]; later, the “low-oxidation-state route”, starting from M(CO)_6_ (M = Mo, W), was employed, which gave rise to the corresponding molybdenum and tungsten benzylidyne complexes [18–20].

In addition to these, several well-defined alkylidyne complexes have been developed and successfully utilized in alkyne metathesis in the past decade. The molybdenum alkylidyne complex with triphenylsiloxide ligands (type **II**) introduced by A. Fürstner and co–workers is frequently used in the total synthesis of natural products [23–27]. A unique catalytic reactivity towards conjugated diynes was observed for the tungsten benzylidyne complex with OSi(O*t-*Bu)_3_ ligands (type **III**) [28–30]. The catalyst **III**, which is capable of promoting conventional alkyne metathesis [19], also proved to be highly active in the cross metathesis of symmetric 1,3-butadiynes to form unsymmetrically substituted 1,3-butadiynes [30]. W. Zhang and his group introduced several multidentate phenoxide ligands to molybdenum propylidyne precursors to form chelate complexes of type **IV** [31–34]. These catalysts were especially successful in the construction of supramolecular materials such as ethynylene-linked polymers [11,35], porous networks [36] and molecular cages [37–43]. Furthermore, living ring-opening alkyne metathesis polymerization (ROAMP) has been intensely studied for different molybdenum alkylidyne complexes by the group of F. R. Fischer, who was able to monitor the influence of both the alkylidyne moiety as well as the ancillary ligands [44–48].

More recently, we expanded the “low-oxidation-state route” to the synthesis of 2,4,6-trimethylbenzylidyne complexes of molybdenum and tungsten [18,49–50], which led to an increased steric demand at the metal center. This proved to be advantageous for the catalytic activity, since the removal of coordinating solvents like THF or DME was facilitated [49]. The molybdenum 2,4,6-trimethylbenzylidyne complex [MesC≡Mo{OC(CF_3_)_2_Me}_3_] (Figure 1, **MoF6**) represents the first alkyne metathesis catalyst capable of effective and highly selective terminal alkyne metathesis [49,51–53]. Later, a study was conducted to determine the optimum degree of fluorination of the alkoxide ligands for tungsten alkylidyne complexes [53–54]. It was found that the tungsten alkylidyne complex [MesC≡W{OC(CF_3_)Me_2_}_3_] (Figure 1, **WF3**) showed excellent catalytic performance not only in the metathesis of internal but also, for the first time with the metal tungsten, terminal alkynes at room temperature [54]. Our studies clearly display a strong dependency of the catalytic alkyne metathesis activity on the metal-alkoxide combination. The electrophilicity of the metal sites can be controlled by the number of fluorine atoms of the ancillary fluoroalkoxide ligands [55–57]. The difference in the optimum degree of fluorination for molybdenum and tungsten is rationalized by the increased intrinsic electrophilicity of tungsten compared to molybdenum [56].

**Scheme 1:**
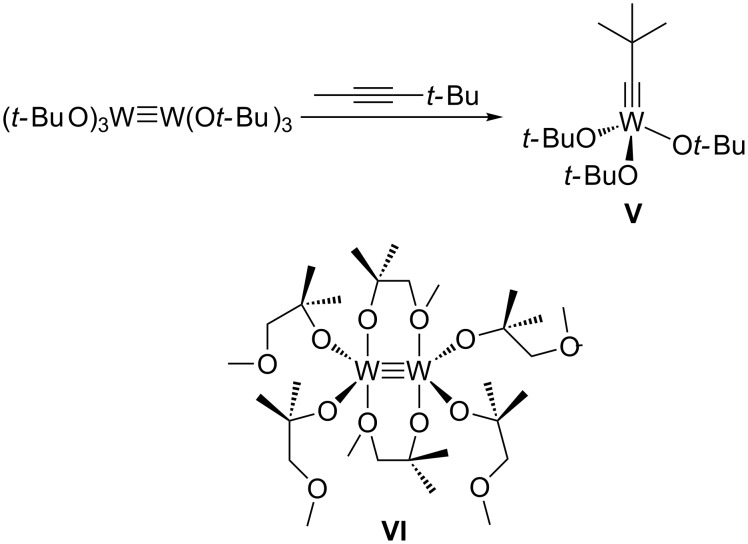
Synthesis of alkylidyne complex **V** from bimetallic [(*t-*BuO)_3_W≡W(O*t-*Bu)_3_]; the catalytically active ditungsten complex [W_2_(MMPO)_6_] (**VI**, MMPO = 1-methoxy-2-methylpropan-2-ol) [68].

Based on these insights into the structure–activity relationship of alkyne metathesis catalysts, we wanted to establish an alternative and convenient access to highly active catalysts. Herein, we report the systematic study on the metathesis performance of bimetallic hexaalkoxide complexes M_2_(OR)_6_ (M = Mo, R = OC(CF_3_)_2_Me, **Mo2F6**; M = W, R = OC(CF_3_)Me_2_, **W2F3**), which draw upon the most catalytically active alkylidyne complexes **MoF6** and **WF3**. R. R. Schrock synthesized the first alkylidyne complex which was able to undergo alkyne metathesis, [*t-*BuC≡W(O*t-*Bu)_3_] (**V**), originally from [NEt_4_][*t-*BuC≡WCl_4_] [22,58–61]. Subsequently, he reported a protocol to synthesize the alkylidyne complex **V** by a stoichiometric alkyne metathesis reaction of the ditungsten complex [(*t-*BuO)_3_W≡W(O*t-*Bu)_3_] with MeC≡C*t-*Bu (Scheme 1) [62]. Even though Schrock’s catalyst **V** was the most established alkyne metathesis catalyst for many years [63–64], it does not promote terminal alkyne metathesis efficiently and leads to polymerization initiated by intermediate deprotiometallacyclobutadiene species [55,60–61,65–67]. Moreover, the bimetallic [(*t-*BuO)_3_W≡W(O*t-*Bu)_3_] complex has not been directly employed in catalytic alkyne metathesis.

A. Mortreux and his group found that the alkyne metathesis selectivity of Schrock’s original catalyst **V** can be enhanced by adding an external ligand like quinuclidine to the reaction mixture [61]. Thereby, the self-metathesis yield of 1-heptyne could be increased to 80% at elevated temperatures. Based on this approach, the dinuclear tungsten complex [W_2_(MMPO)_6_] (**VI**, MMPO = 1-methoxy-2-methylpropan-2-ol) was isolated which catalyzed alkyne metathesis of 1-heptyne at elevated temperatures [68] and to date represents the only well-defined ditungsten complex which has been successfully used in alkyne metathesis.

The organometallic chemistry of the M_2_X_6_ complexes (X = R (alkyl), NR_2_, OR) with metal-metal triple bonds (M = Mo, W) has attracted attention for many years (mainly during the 70s, 80s and 90s) [69]. A plethora of dinuclear compounds has been published [70–78], and detailed studies on their reactivity have been conducted [79–85]. Therefore, the reactivity of ditungsten complexes towards alkynes has been known for quite some time. The metal–metal triple bond of many ditungsten complexes can be cleaved by alkynes in a metathesis-like reaction to form the corresponding alkylidyne complexes [62,86]. Dinuclear Mo≡Mo complexes, however, have not yet been cleaved efficiently by alkynes [87].

## Results and Discussion

### Complex synthesis

**Scheme 2:**
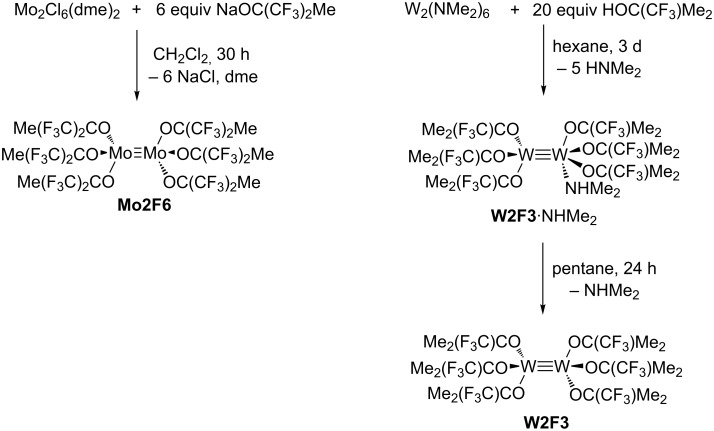
Synthesis of hexakis(fluoroalkoxide) dimers **Mo2F6** [73] and **W2F3**.

The dimeric molybdenum complex [Mo_2_Cl_6_(dme)_2_] (dme = 1,2-dimethoxyethane) serves as an excellent starting material for compounds of the type Mo_2_X_6_ (X = alkyl, alkoxide) [73]. The desired hexakis(fluoroalkoxide) dimer [Mo_2_{OC(CF_3_)_2_Me}_6_] (**Mo2F6**) was first isolated by D. Rogers and his group by salt metathesis of [Mo_2_Cl_6_(dme)_2_] with 6 equiv of NaOC(CF_3_)_2_Me (Scheme 2) [73]. This reaction affords a red, sparingly soluble complex in moderate yield (28%).

**Figure 2:**
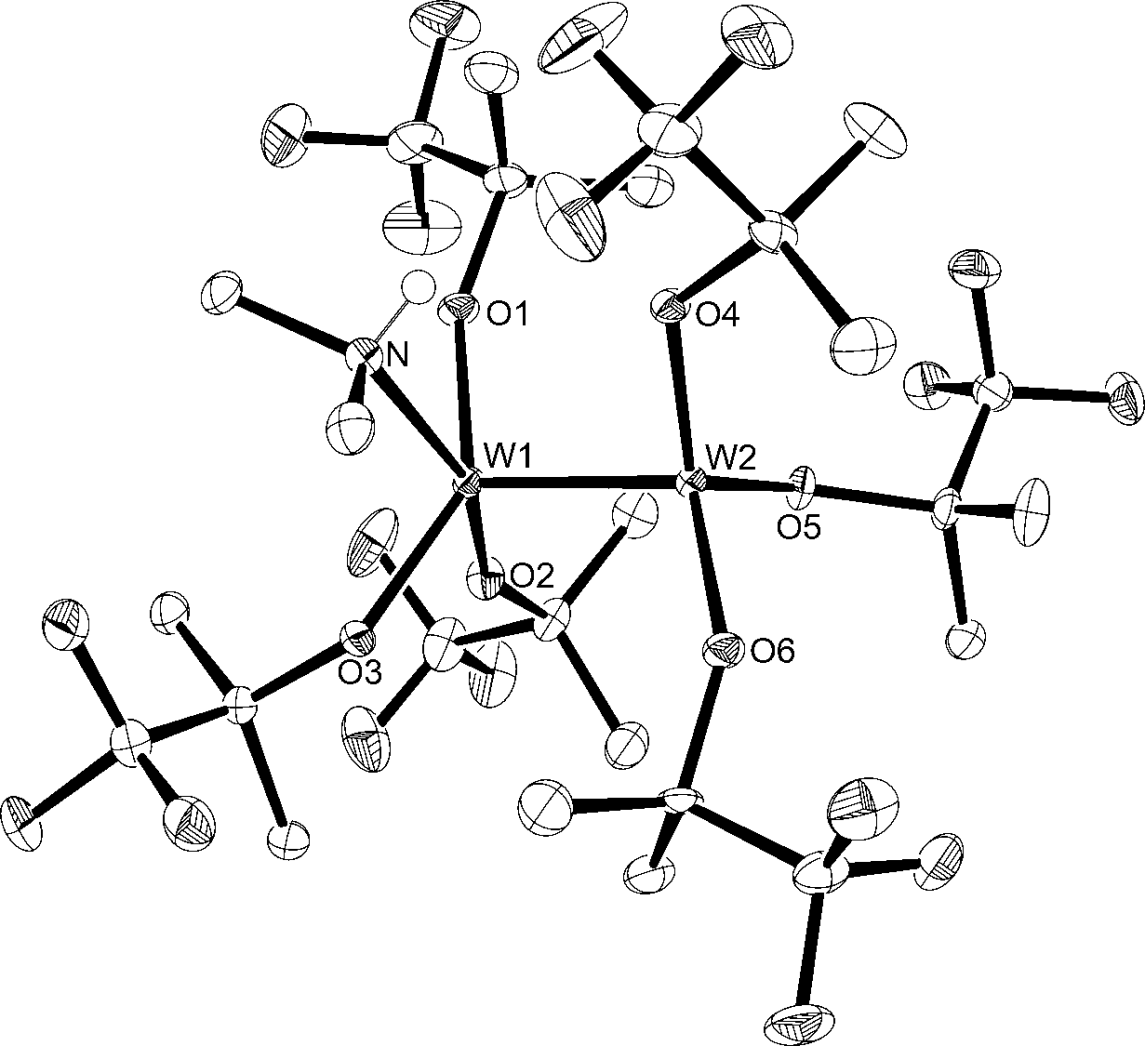
Molecular structure of **W2F3**·NHMe_2_ with thermal displacement parameters drawn at 50% probability. Hydrogen atoms are omitted for clarity.

The bimetallic tungsten analogue to **WF3**, [W_2_{OC(CF_3_)Me_2_}_6_] (**W2F3**), can be prepared by the reaction of [NaW_2_Cl_7_(THF)_5_] with 6 equiv of NaOC(CF_3_)Me_2_ [86], but this procedure requires one equivalent of toxic sodium amalgam. Therefore, we decided to attempt the protonolysis of hexakis(dimethylamido)ditungsten [W_2_(NMe_2_)_6_] with the alcohol HOC(CF_3_)Me_2_ [71,88], which has been described very briefly in the literature [89]. [W_2_(NMe_2_)_6_] is easily accessible from WCl_4_ and LiNMe_2_ and has emerged as an important starting material for various dinuclear tungsten compounds [90]. M. H. Chisholm and co-workers used 6 equiv of the free alcohol to exchange the amide ligands and isolated the bis(dimethylamino) adduct of the ditungsten complex [89]. The amine ligands were liberated under reduced pressure and at elevated temperature. However, in our hands, an excess of HOC(CF_3_)Me_2_ was required to drive the reaction to completion and led to the formation of the complex [W_2_{OC(CF_3_)Me_2_}_6_(NHMe_2_)] (**W2F3**·NHMe_2_) (Scheme 2). The additional amine ligand stems from the protonolysis reaction of the amide with the fluorinated alcohol. Brown crystals suitable for X-ray diffraction analysis were isolated from a saturated pentane solution at −40 °C. The molecular structure of this complex was established by X-ray diffraction analysis. The ORTEP diagram is shown in Figure 2, and selected bond lengths and angles are displayed in Table 1.

**Table 1:** Selected bond lengths [Å] and angles [°]:

Bond	Bond length [Å]	Bond angle	Angle [°]	Bond angle	Angle [°]

W1–W2	2.3452(2)	O1–W1–O2	93.75(11)	O5-W2-O4	122.91(11)
W1–O1	1.905(2)	O1–W1–O3	145.01(10)	O1–W1–W2	104.75(8)
W1–O2	1.911(3)	O2–W1–O3	91.61(11)	O2–W1–W2	108.97(8)
W1–O3	1.970(2)	O1–W1–N	82.15(11)	O3–W1–W2	106.06(7)
W1–N	2.270(3)	O2–W1–N	159.81(11)	N–W1–W2	91.17(8)
W2–O4	1.930(2)	O3–W1–N	81.06(11)	O4–W2–W1	98.33(7
W2–O5	1.818(2)	O6–W2–O5	110.53(11)	O5–W2–W1	99.29(7)
W2–O6	1.872(2)	O6–W2–O4	112.06(11)	O6–W2–W1	111.85(8)

The tungsten–tungsten triple bond of 2.3452(2) Å falls in the range of previously reported bond lengths of this type [69]. For example, the W≡W distance in [W_2_{OC(CF_3_)_2_Me}]_6_ is 2.309(3) Å [78], 2.430(8) Å in [W_2_(MMPO)_6_] [68], and 2.332(1) Å in [W_2_(OCHMe_2_)_6_(py)_2_] (py = pyridine) [90]. The W1–N bond length of 2.270(3) Å is longer compared to the W–N distances in [W_2_(NMe_2_)_6_] (1.95(2)–1.99(2) Å) [71] and [W_2_Cl_2_(NMe_2_)_4_] (1.935(8)–1.937(9) Å) [91]. This is attributed to the neutral nature of the NHMe_2_ ligand compared to the negative NMe_2_ ligand and indicates a weak bond between the tungsten and the nitrogen atom. Coordination of the NHMe_2_ ligand to W1 affords markedly longer W1–O bonds (1.905(2)–1.970(2) Å) than the W2–O bonds (1.818(2)–1.930(2) Å). The W1 atom is coordinated in an almost square-pyramidal fashion (τ_5_ = 0.25) [92] while the W2 atom adopts a nearly tetrahedral geometry (τ_4_ = 0.89) [93], which is usually observed in X_3_W≡WX_3_ complexes [72,83,94]. The coordination of one amine ligand after the protonolysis of the amide ligands in [W_2_(NMe_2_)_6_] has been observed before, e.g., in [W_2_(OAr)_6_(HNMe_2_)] (Ar = 3,5-dimethylphenoxide) [95].

Complex **W2F3**·NHMe_2_ appeared to be rather unstable especially in hexane and pentane solutions. Over a period of 24 h, a color change from brown to bright red occurred in solution. This observation indicates the loss of the additional amine ligand, and after recrystallization from pentane at −25 °C, the red complex **W2F3** was isolated. The ^1^H NMR spectrum reveals the only expected signal at 1.51 ppm, which is in line with the previously reported values, where **W2F3** had been prepared from [NaW_2_Cl_7_(THF)_5_] [86]. The ^13^C and ^19^F NMR spectra are also consistent with literature values. Crystals of **W2F3** suitable for X-ray diffraction analysis were obtained upon cooling a saturated pentane solution to −40 °C. Unfortunately, the crystal structure suffers from severe disorder. Each tungsten atom is disordered over four positions, and additionally, the alkoxide ligands are also disordered (for more details, see Supporting Information File 1). Therefore, the crystal structure only confirms the connectivity and does not allow the discussion of bond lengths and angles. This disorder pattern has been reported repeatedly for molybdenum and tungsten hexaalkoxides and silanolates [73,78,83,96–98]. An ORTEP diagram of **W2F3** is displayed in Supporting Information File 1 (Scheme S13).

As stated above, R. R. Schrock could generate alkyne metathesis catalysts of type **V** (Scheme 1) from the corresponding bimetallic complex [62,99]. Thus, we attempted the cleavage of the M≡M bond of **Mo2F6** and **W2F3** by an alkyne to generate the corresponding benzylidyne complexes. Unfortunately, as reported in the past by Schrock [62,87], we could not achieve the selective cleavage of the triple bond in **Mo2F6** by internal or terminal alkynes to isolate the corresponding alkylidyne complex. In an NMR study on the cleavage of the Mo≡Mo triple bond, in which **Mo2F6** was treated with two equivalents of 1-phenyl-1-propyne, no signals corresponding to a possible molybdenum alkylidyne complex were detected in the ^1^H and ^19^F NMR spectra over a period of three days.

**Scheme 3:**
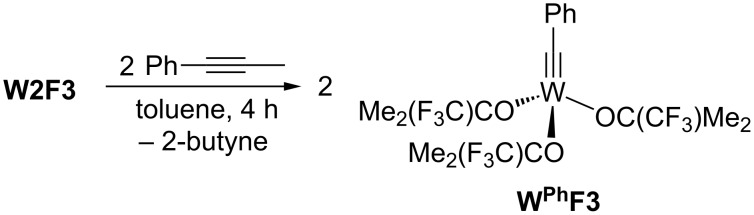
Preparation of the alkylidyne complex **W^Ph^F3**.

In contrast, the reaction of the bimetallic tungsten complex **W2F3** with two equivalents of 1-phenyl-1-propyne in toluene afforded the light yellow benzylidyne complex **W^Ph^F3** (Scheme 3) in satisfactory yield after recrystallization from *n*-pentane. In a metathesis-like reaction, the W≡W bond is cleaved, with 2-butyne forming as a side product. Following this reaction by ^1^H and ^19^F NMR spectroscopy revealed fast and selective formation of **W^Ph^F3**, and after 14 minutes, most of the starting material **W2F3** was already consumed, with full conversion observed after 28 minutes. Selected ^19^F NMR spectra can be found in Figure S7 of Supporting Information File 1.

**Figure 3:**
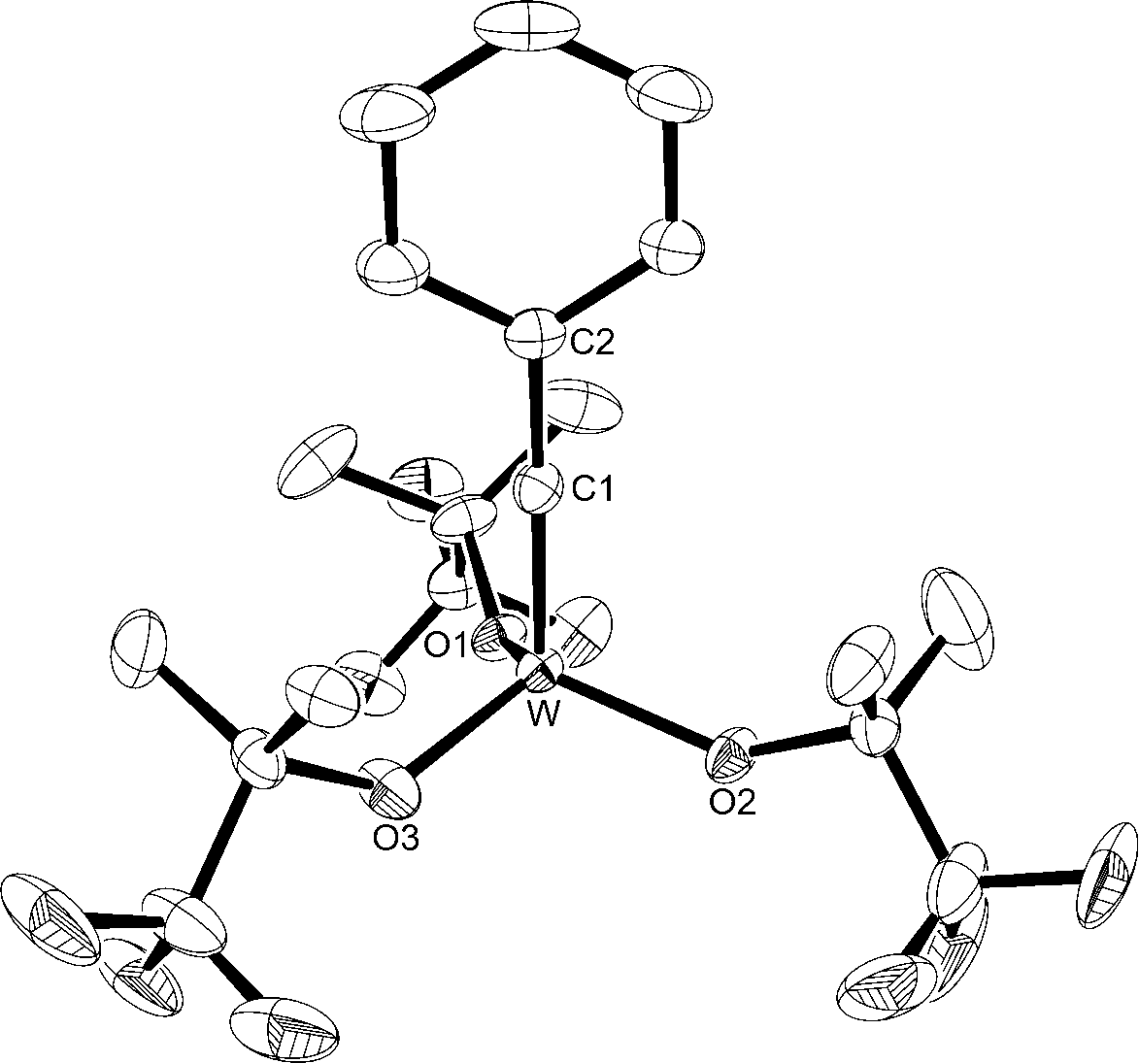
Molecular structure of **W^Ph^F3** with thermal displacement parameters drawn at 50% probability. Hydrogen atoms and minor components of the disordered OC(CF_3_)Me_2_ groups are omitted for clarity.

The ^1^H NMR spectrum of **W^Ph^F3** displays two multiplets in the aromatic region for the benzylidyne hydrogen atoms and one singlet for the methyl groups of the trifluoro-*tert*-butoxy ligand at 1.65 ppm. In the ^13^C NMR spectrum, the signal of the carbyne carbon atom can be found at 266.9 ppm, which is in the range typically observed for RC≡W moieties [16–18,49–50,54]. The ^19^F NMR spectrum only exhibits one singlet for the complex with a chemical shift of −82.4 ppm. Crystals suitable for X-ray diffraction analysis were isolated from a saturated pentane solution at −40 °C; again, the crystal structure suffers from crystallographic problems: one alkoxide ligand is refined on two positions, while another one is refined on three positions. The crystal structure, which is displayed in Figure 3, only confirms the connectivity of this molecule, and discussion of any bond length is not meaningful (for more details, see Supporting Information File 1). A similar crystal structure with the 2,4,6-trimethylbenzylidyne moiety at the tungsten atom, which does not exhibit disorder, has been reported previously [54].

### Catalytic studies

With the bimetallic complexes **Mo2F6** and **W2F3** and the new alkylidyne complex **W^Ph^F3** at hand, we aimed at systematically investigating the catalytic activity of those complexes. Even though we failed in selectively cleaving the Mo≡Mo triple bond, we attempted catalytic alkyne metathesis with **Mo2F6**. Interestingly, a marginal catalytic activity could be detected for complex **Mo2F6**: over the course of four days, the dimolybdenum complex achieved a conversion of 70% in the self-metathesis of the standard substrate 3-pentynyl benzyl ether in toluene in the presence of molecular sieves (MS 5 Å) as 2-butyne scavenger. The conversion versus time diagram (Supporting Information File 1, Figure S8) exhibits a sigmoidal curve progression, which indicates the slow formation of a catalytically active species, presumably an alkylidyne complex, despite our inability to monitor the formation of such a species by NMR spectroscopy. We attribute the poor catalytic performance of **Mo2F6** to the low solubility in all common solvents [73], since most of the compound still remained undissolved in the reaction mixture after four days. However, all efforts to optimize the metathesis conditions and to achieve higher conversions failed. Attempts to increase the solubility of **Mo2F6** in toluene by performing the metathesis reaction at 60 °C led to no detectable conversion of the starting material. Furthermore, catalysis in CH_2_Cl_2_ afforded even lower conversions compared to toluene, while the metathesis failed completely in diethyl ether.

**Scheme 4:**
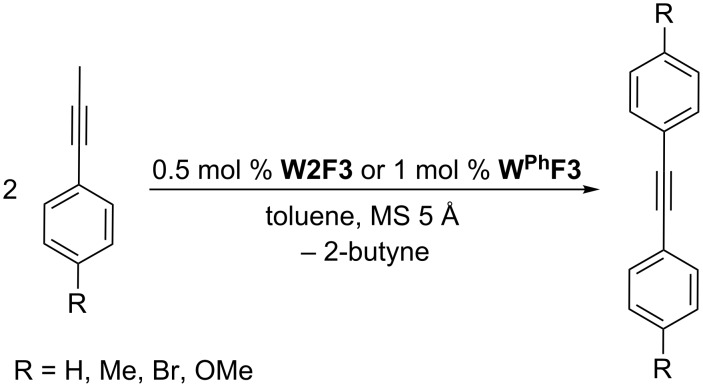
Self-metathesis of 1-phenyl-1-propyne derivatives by tungsten complexes **W2F3** and **W^Ph^F3**.

**Figure 4:**
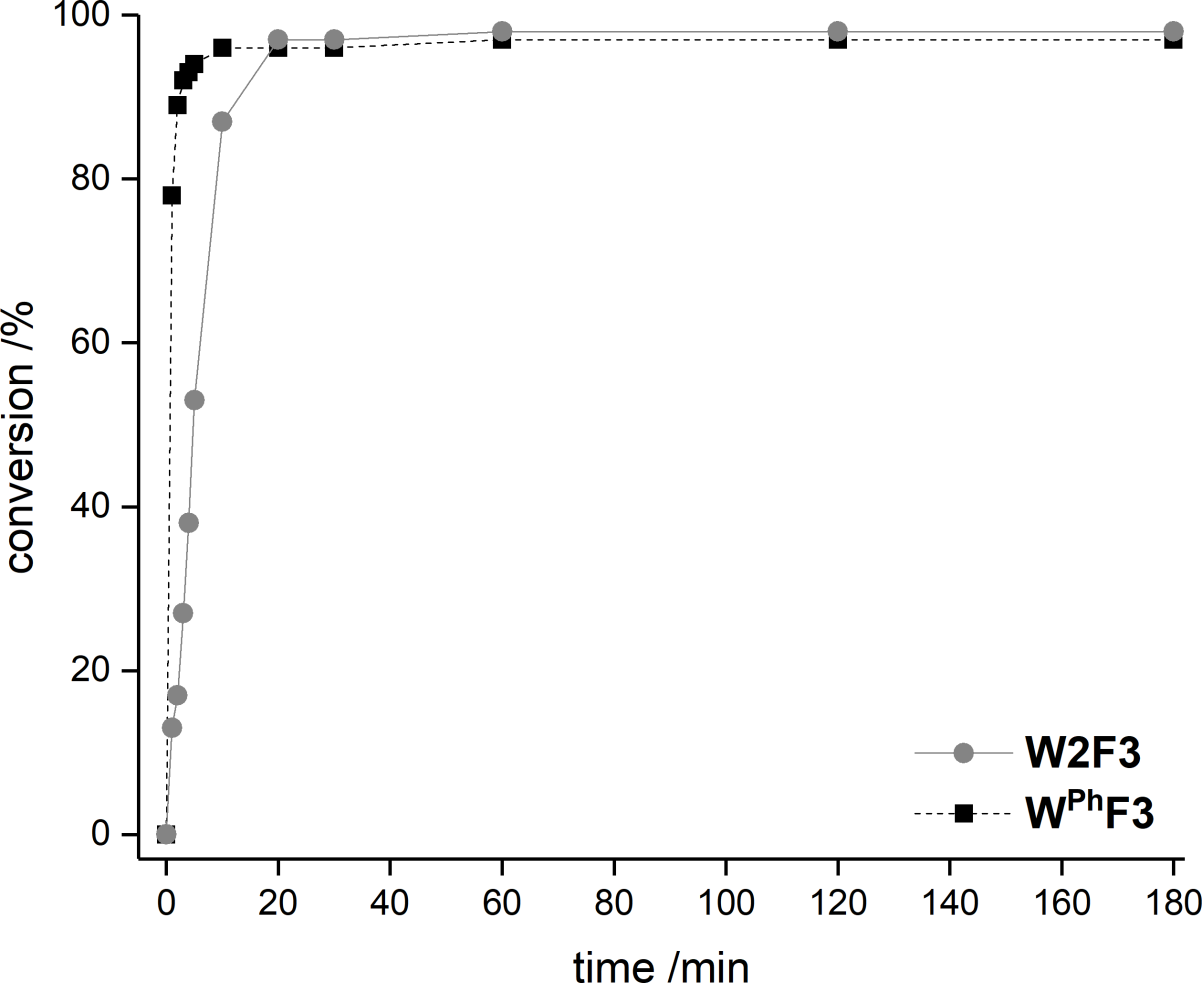
Conversion versus time diagram for the self-metathesis of 1-phenyl-1-propyne catalyzed by 0.5 mol % **W2F3** (grey) and 1 mol % **W^Ph^F3** (black).

For the potential tungsten catalysts, toluene solutions of 1-phenyl-1-propyne were treated with **W2F3** (0.5 mol %) and **W^Ph^F3** (1 mol %) and stirred in the presence of molecular sieves (MS 5 Å) as 2-butyne scavenger and *n*-decane as internal standard at room temperature (Scheme 4). The catalysis was initially monitored over time through gas chromatography, affording the conversion versus time diagram depicted in Figure 4.

Figure 4 clearly shows that both tungsten complexes are active in the metathesis of 1-phenyl-1-propyne, with the bimetallic compound **W2F3** (grey) showing a slower initiation rate compared to the alkylidyne complex **W^Ph^F3**. For the bimetallic complex **W2F3**, an additional initiation step is required, in which the W≡W triple bond is cleaved and catalytically active alkylidyne species are formed. Therefore, the conversion of the substrate with catalyst **W2F3** is significantly slower at the beginning of the reaction. The initial catalyst turnover frequencies were calculated from the conversion of 1-phenyl-1-propyne after one minute (TOF_1min_). The TOF of the alkylidyne complex **W^Ph^F3** reaches 78 min^−1^ (1.30 s^−1^), while **W2F3** has a significantly lower TOF of 13 min^−1^ (0.21 s^−1^) after one minute; this value is based on the formation of two catalytically active alkylidyne species upon treatment with the alkyne substrate. After 10 minutes, the alkylidyne complex **W^Ph^F3** has accomplished nearly full conversion and after 20 minutes, catalyst **W2F3** achieves the same conversion of the starting material. The maximum conversion of around 97% is reached for both catalysts within 60 minutes. Accordingly, we aimed at further monitoring the substrate scope of the complexes including the metathesis of terminal alkynes as well as ring-closing alkyne metathesis (RCAM).

Table 2 summarizes the isolated yields for various self-metathesis and RCAM reactions. These findings are in line with our initial results regarding the conversion of 1-phenyl-1-propyne (Table 2, entry 1). Both tungsten complexes afforded excellent yields in the metathesis of different *para*-substituted phenylpropynes (Table 2, entries 2–4). For both catalysts, the yields are identical within the error of the experiment. Furthermore, the well-established substrates 3-pentynyl (R = Me) and 3-butynyl (R = H) benzyl ether (Table 2, entry 5) and 3-pentynyl (R = Me) and 3-butynyl (R = H) benzyl ester (Table 2, entry 6) afforded good isolated yields. The bimetallic complex **W2F3** is even capable of metathesizing terminal alkynes at room temperature and performs in the same manner as the alkylidyne complex **W^Ph^F3**. Additional conversion versus time diagrams are displayed in Figures S9 and S10 in Supporting Information File 1. Finally, the ditungsten catalyst **W2F3** was also employed in alkyne cross-metathesis (ACM), a reaction which is of large interest for the application of alkyne metathesis, but often leads to product mixtures. Therefore, (trimethylsilyl)propyne and (trimethylsilyl)acetylene were chosen as reaction partners in ACM, since this reaction proved to be quite efficient in the past [25,54,100]. A toluene solution of both substrates (1:2 ratio, TMS–alkyne in excess) was charged with the catalyst **W2F3** (0.5–1 mol %) in the presence of molecular sieves 5 Å and stirred for two hours at room temperature. The isolated yields of the ACM are summarized in Table 3. The depicted reactions selectively afforded the unsymmetrical alkynes, corroborating that the bimetallic tungsten complex **W2F3** is able to introduce a trimethylsilyl protecting group to alkynes.

**Table 2:** Alkyne metathesis of different substrates.^a^

Entry	Substrate	Product	Cat.	R	Yield [%]

1	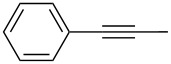	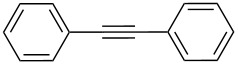	**W2F3** **W^Ph^F3**		96 95
2	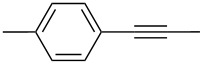	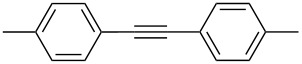	**W2F3** **W^Ph^F3**		95 96
3	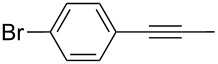	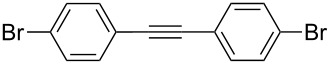	**W2F3** **W^Ph^F3**		94 97
4	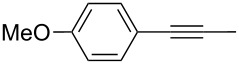	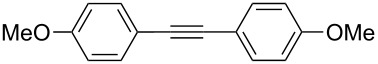	**W2F3** **W^Ph^F3**		98 97
5	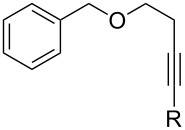	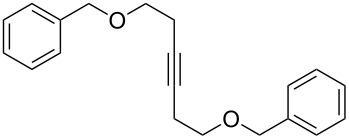	**W2F3**	R = Me R = H	96 88
			**W^Ph^F3**	R = Me R = H	94 88
6	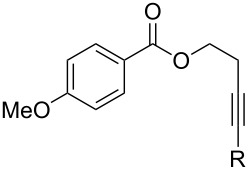	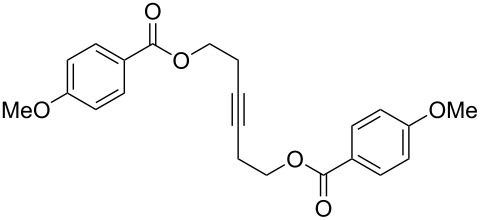	**W2F3**	R = Me R = H	93 79
			**W^Ph^F3**	R = Me R = H	94 72
7	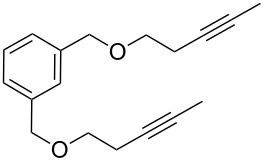	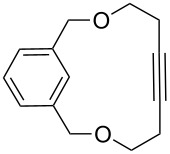	**W2F3** **W^Ph^F3**		86 84
8	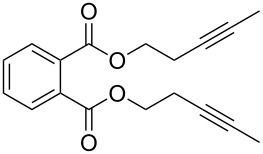	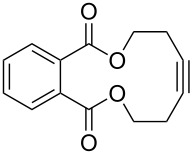	**W2F3** **W^Ph^F3**		93 96

^a^Self-metathesis: substrate (0.5 mmol), catalyst (0.5 mol % **W2F3**; 1 mol % **W^Ph^F3**), toluene (internal alkynes: 2.5 mL, 200 mM; terminal alkynes: 24 mL, 21 mM), MS 5 Å (500 mg), 25 °C, 2 h. RCAM: substrate (0.5 mmol), catalyst (1 mol % **W2F3**; 2 mol % **W^Ph^F3**), toluene (24 mL, 21 mM), MS 5 Å (1.0 g), 25 °C, 2 h.

**Table 3:** Alkyne cross metathesis (ACM) with catalyst **W2F3**.^a^

Entry	Substrates		Product	Cat.	Yield [%]

1	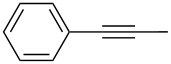	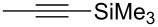	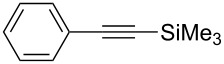	0.5 mol %	87
2	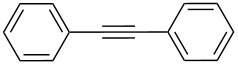	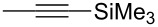	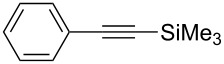	1 mol %	82
3	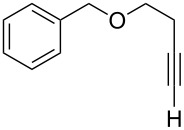	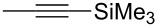	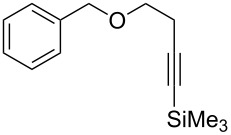	0.5 mol %	93
4		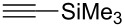		0.5 mol %	92

^a^Substrate (0.5 mmol), TMS-propyne or TMS-acetylene (1 mmol), toluene (internal alkynes: 2.5 mL, terminal alkynes: 24 mL), MS 5 Å (500 mg), 25 °C, 2 h.

## Conclusion

Previously, we have reported the optimum level of fluorination in **MoF6** and **WF3** as the most catalytically active alkylidyne complexes [54]. Thus, we intended to study the catalytic activity of the well-known bimetallic molybdenum and tungsten complexes bearing the same fluoroalkoxide ligands. Indeed, **W2F3** as the bimetallic analogue to mononuclear **WF3** is highly active in the metathesis of internal and even terminal alkynes and also promotes alkyne cross-metathesis efficiently. The **Mo2F6** complex, however, does not exhibit satisfactory alkyne metathesis activity, which we attribute to its low solubility. Furthermore, the corresponding mononuclear benzylidyne complex **W^Ph^F3** is easily accessible from the dinuclear **W2F3** complex and performs equally well compared to the latter.

The finding that **W2F3** is a highly active alkyne metathesis pre-catalyst and does not have to be converted into an alkylidyne species prior to catalysis could be beneficial for future applications of alkyne metathesis since this protocol represents a convenient approach to alkyne metathesis catalysts in two steps starting from WCl_4_.

## Supporting Information

CCDC 1850924−1850926 contain the supplementary crystallographic data for this paper. These data can be obtained free of charge via http://www.ccdc.cam.ac.uk/data_request/cif.

File 1 Experimental section, NMR spectra, catalysis procedure and product characterization, crystallographic details for **W2F3**·(NHMe_2_), **W2F3** and **W^Ph^F3**.
