# Supplementary material for: Efficient catalytic alkyne metathesis with a fluoroalkoxy-supported ditungsten(III) complex
Source: Beilstein J Org Chem. 2018 Sep 18;14:2425–34. doi: 10.3762/bjoc.14.220 (PMC6178283; doi:10.3762/bjoc.14.220)
Supplement: File 1 — Experimental section, NMR spectra, catalysis procedure and product characterization, crystallographic details for W2F3·(NHMe2), W2F3 and WPhF3. [file Beilstein_J_Org_Chem-14-2425-s001.pdf]

Supporting Information

for

Efficient catalytic alkyne metathesis with a fluoroalkoxy-supported ditungsten(III) complex

Henrike Ehrhorn[1], Janin Schlösser[1], Dirk Bockfeld[1] and Matthias Tamm*[1]

Address: [1]Institut für Anorganische und Analytische Chemie, Technische Universität Braunschweig, Hagenring 30, 38106 Braunschweig, Germany

Email: Matthias Tamm - m.tamm@tu-bs.de

*Corresponding author

Experimental section, NMR spectra, catalysis procedure and product characterization, crystallographic details for W2F3·($NHMe_2$), W2F3 and WPhF3

Experimental section S1
- General experimental considerations S1
- Analytical methods S1
- Experimental procedures S1
- NMR Spectra S4

Catalytic studies S6
- Comparison of catalytic activities. S6
- General procedure for self-metathesis S7
- General procedure for RCAM S7
- General Procedure for ACM S7
- Catalysis products S7
- Conversion versus time diagrams S9

X-ray structure analysis S10

References S15

Experimental section

General experimental considerations

All operations with air and moisture sensitive compounds were conducted in a glove box (MBraun 200B) under dry and oxygen-free argon atmosphere or on a Schlenk line using standard Schlenk techniques. Solvents were purified and dried by a MBraun GmbH solvent purification system and stored over molecular sieve (3−4 Å). **Mo2F6**[1], $[W_2(NMe_2)_6]$[2], $NaOC(CF_3)_2Me$[3] and catalysis substrates 3-pentynyl benzyl ether[4], 3-pentynyl benzyl ester[4], 3-butynyl benzyl ether[5], 3-butynyl benzyl ester[5], *m*-xylene-di(oxapent-3-yne)[5], *o*-xylylene-di(oxapent-3-yne)[6] were synthesized according to literature methods. The syntheses of *para*-substituted phenylpropynes were based on the literature procedure by Y. Tsuji[7]. All other compounds were obtained from commercial sources (Sigma-Aldrich, abcr, Acros, TCI, Alfa Aeser, flurorochem) and were used without further purification. Molecular sieves MS 5 Å (Sigma-Aldrich, powder <50 micrometer) for metathesis reactions were dried for 24 h at 180 °C under vacuum prior to use. 1-Phenyl-1-propyne was distilled over CaH_2 and filtered through alumina (Al_2O_3) at least three times and additionally prior to use in catalysis.

Analytical methods

^1H, ^{19}F and ^{13}C NMR spectra were recorded on Bruker AVIIIHD-300, AVIII-400 and AVII-600 instruments at room temperature. Chemical shifts (δ) are expressed in ppm (parts per million). ^1H and ^{13}C NMR spectra are referenced to residual solvent signals (C_6D_6: δ(H) 7.16 ppm, δ(C) 128.06 ppm; $CDCl_3$: δ(H) 7.26 ppm, δ(C) 77.16 ppm; CD_2Cl_2: δ(H) 5.32 ppm, δ(C) 53.84 ppm). For ^{19}F NMR, an external calibration with $CFCl_3$ was used. Coupling constants (*J*) are reported in Hertz (Hz). Multiplicities are expressed with s (singlet), d (doublet), t (triplet), q (quartet), or m (multiplet). The number of protons (n) for a given resonance is indicated by nH. When necessary, signal assignment was confirmed by two-dimensional H,C-HSQC NMR and H,C-HMBC NMR experiments. Elemental analyses were performed by combustion and gas chromatographic analysis using a VarioMICRO Tube and WLD detection. Gas chromatography (GC) was executed on a Hewlett Packard 5890 SERIES II using a DB5-HT column and FID detection. For calibration *n*-decane was used as an internal standard.

Experimental procedures

$[Mo_2\{OC(CF_3)_2Me\}_6]$ (Mo2F6)[1]

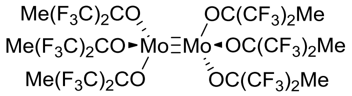

A solution of 400 mg (0.68 mmol) $[Mo_2Cl_6(dme)_2]$[1] in 25 mL CH_2Cl_2 was treated with 933 mg (4.08 mmol) $NaOC(CF_3)_2Me$ over a period of 15 minutes. The reaction mixture was stirred for 30 hours. Afterwards the solvent was removed under reduced pressure and the residue was extracted with Et_2O (1 × 20 mL, 2 × 10 mL). The combined extracts were filtered over a patch of Celite®. The saturated ether solution was stored at −25 °C whereupon the product crystallized as red crystals (243 mg, 0.19 mmol, 28%).

^{1}H-NMR (300.3 MHz, C_6D_6, 300 K): δ [ppm] = 1.44 [s, $C(CF_3)_2(CH_3)$]. **^{19}F{^{1}H}-NMR** (282.5 MHz, C_6D_6, 300 K): δ [ppm] = −77.5 [s, CF_3]. Anal. Calcd. for $C_{24}H_{18}F_{36}O_6Mo_2$: C 22.55, H 1.42. Found: C 22.51, H 1.44.

[W_2{OC(CF_3)Me_2}$_6$($NHMe_2$)] (**W2F3**·$NHMe_2$)

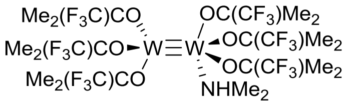

A solution of 494 mg (0.78 mmol) [$W_2(NMe_2)_6$] in 20 mL hexane was treated with 2.0 g (15.6 mmol) HOC(CF_3)Me_2. Upon addition of the alcohol the reaction mixture turned dark brown. After three days, the solvent was removed under reduced pressure. The dark residue was treated with $CHCl_3$ (30 mL) and the resulting suspension was filtered over a patch of Celite®. The filtrate was concentrated and stored at −25 °C whereupon the product crystallized as dark brown crystals (595 mg, 0.506 mmol, 65%). Anal. Calcd. for $C_{26}H_{43}F_{18}NO_6W_2$: C 26.57, H 3.69, N 1.19. Found: C 26.57, H 3.11, N 1.33.

[W_2{OC(CF_3)Me_2}$_6$] (**W2F3**)

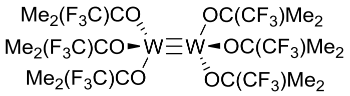

100 mg (0.085 mmol) **W2F3**·$NHMe_2$ were dissolved in pentane (7 mL). The solution was stirred for 24 h at room temperature and the amine-free product crystallized from the saturated solution at −25 °C as bright red crystals (70 mg, 0.062 mmol, 73%).

^{1}H-NMR (300.3 MHz, C_6D_6, 300 K): δ [ppm] = 1.51 [s, $C(CF_3)(CH_3)_2$]. **^{13}C{^{1}H}-NMR** (75.5 MHz, C_6D_6, 300 K): δ [ppm] = 126.9 [q, $^{1}J_{CF}$ = 285 Hz, $C(CF_3)(CH_3)_2$], 83.0 [q, $^{2}J_{CF}$ = 29 Hz, $C(CF_3)(CH_3)_2$], 25.3 [s, $C(CF_3)(CH_3)_2$]. **^{19}F{^{1}H}-NMR** (282.5 MHz, C_6D_6, 300 K): δ [ppm] = −82.7 [s, CF_3]. Anal. Calcd. for $C_{24}H_{36}F_{18}O_6W_2$: C 25.51, H 3.21. Found: C 25.19, H 3.12.

[PhC≡W{OC(CF_3)Me_2}$_3$] (**W^{Ph}F3**)

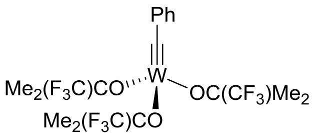

A solution of 170 mg (0.15 mmol) **W2F3** in 10 mL toluene was treated with 35 mg (0.30 mmol) 1-phenyl-1-propyne. The solution was stirred for 4 h at room temperature and afterwards the solvent was removed under reduced pressure. The light brown residue was taken up in pentane and filtered over a short patch of Celite®. Cooling of a saturated solution to −25 °C lead to the formation of light yellow crystals (113 mg, 0.17 mmol, 58%).

^{1}H-NMR (600.1 MHz, CD_2Cl_2, 300 K): δ [ppm] = 7.35–7.37 [m, 2H, CH], 7.04 [d, 2H, $^{3}J_{HH}$ = 7.4 Hz, CH], 7.02 [t, 1H, $^{3}J_{HH}$ = 7.4 Hz CH], 1.65 [s, 18H, CH_3]. **^{13}C{^{1}H}-NMR** (150.9 MHz, CD_2Cl_2, 300 K): δ [ppm] = 266.9 [s, W≡C], 146.2 [s, *ipso*-C], 132.6 [s, PhC], 127.9 [s, PhC], 127.9 [s, *para*-C], 127.0 [q, $^{1}J_{CF}$ = 285 Hz, $C(CF_3)Me_2$], 83.2 [q, $^{2}J_{CF}$ = 29 Hz, $C(CF_3)Me_2$], 25.5 [s, CH_3]. **^{19}F{^{1}H}-NMR** (282.5 MHz, C_6D_6, 300 K): δ [ppm] = −82.4 [s, CF_3]. Anal. Calcd. for $C_{19}H_{23}F_9O_3W$: C 34.88, H 3.54. Found: C 34.41, H 3.54.

4-Methyl-1-propinylbenzene[7]

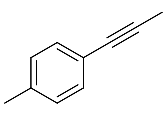

4.65 g (40 mmol) 1-Methyl-4-ethynylbenzene dissolved in 140 mL THF were treated with 50 mL (80 mmol, 1.6 M in hexane) *n*-BuLi at −20 °C. The reaction mixture was stirred for one hour at low temperature and 7.2 mL (14.2 g, 100 mmol) methyl iodide were added dropwise at −20 °C. The solution was warmed to room temperature and stirred for six hours. The reaction was quenched with a saturated NH_4Cl solution (100 mL) and the aqueous phase was extracted with ethyl acetate (3 × 100 mL). The combined organic phases were dried over Na_2SO_4 and the solvent was removed in vacuo. The colorless liquid was distilled at 3 mbar and 65 °C (3.38 g, 26 mmol, 65%).

^{1}H-NMR (300.1 MHz, $CDCl_3$, 300 K): δ [ppm] = 7.27–7.30 [m, 2H, Ar-C*H*], 7.07–7.10 [m, 2H, Ar-C*H*], 2.33 [2, 3H, PhC*H*$_3$], 2.04 [s, 3H, C≡C*H*$_3$]. **^{13}C{^{1}H}-NMR** (75.5 MHz, $CDCl_3$, 300 K): δ [ppm] = 137.6 [s, Ar-*C*], 131.5 [s, Ar-*C*H], 129.1 [s, Ar-*C*H], 121.1 [s, Ar-*C*], 85.1 [s, *C*≡CCH$_3$], 79.9 [s, C≡*C*CH$_3$], 21.5 [s, Ph*C*H$_3$], 4.5 [s, C≡C*C*H$_3$].

4-Bromo-1-propinylbenzene[7]

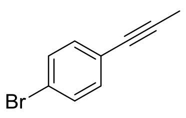

2.0 g (11.1 mmol) 1-Bromo-4-ethynylbenzene dissolved in 60 mL THF were treated with 6.9 mL (11.1 mmol, 1.6 M in hexane) *n*-BuLi at −78 °C. The reaction mixture was stirred for one hour at low temperature and 1.6 mL (3.14 g, 22.1 mmol) methyl iodide were added dropwise at −78 °C. The solution was warmed to room temperature and stirred for three hours. The reaction was quenched with a saturated NH_4Cl solution (50 mL) and the aqueous phase was extracted with CH_2Cl_2 (3 × 80 mL). The combined organic phases were dried over $MgSO_4$ and the solvent was removed in vacuo. After flash chromatography (hexane) the product was isolated as a colorless oil (1.53 g, 7.8 mmol, 71%).

^{1}H-NMR (600.1 MHz, $CDCl_3$, 300 K): δ [ppm] = 7.38–7.43 [m, 2H, Ar-C*H*], 7.21–7.26 [m, 2H, Ar-C*H*], 2.03 [s, 3H, C≡C*H*$_3$]. **^{13}C{^{1}H}-NMR** (150.9 MHz, $CDCl_3$, 300 K): δ [ppm] = 133.1 [s, Ar-*C*H], 131.6 [s, Ar-*C*H], 123.1 [s, Ar-*C*], 121.7 [s, Ar-*C*], 87.3 [s, *C*≡CCH$_3$], 78.9 [s, C≡*C*CH$_3$], 4.5 [s, C≡C*C*H$_3$].

4-Methoxy-1-propinylbenzene[7]

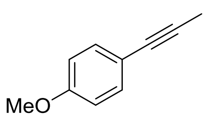

500 mg (3.78 mmol) 1-Methoxy-4-ethynylbenzene dissolved in 15 mL THF were treated with 2.4 mL (3.78 mmol, 1.6 M in hexane) *n*-BuLi at −20 °C. The reaction mixture was stirred for one hour at low temperature and 0.6 mL (7.56 mmol) methyl iodide were added dropwise at −20 °C. The solution was warmed to room temperature and stirred for 16 hours. The reaction was quenched with a saturated NH_4Cl solution (30 mL) and the aqueous phase was extracted with CH_2Cl_2 (3 × 70 mL). The combined organic phases were dried over $MgSO_4$ and the solvent was removed in vacuo. After flash chromatography (hexane) the product was isolated as a colorless oil (475 mg, 3.25 mmol, 86%).

^{1}H-NMR (400.4 MHz, $CDCl_3$, 300 K): δ [ppm] = 7.31–7.34 [m, 2H, Ar-C*H*], 6-79–6.83 [m, 2H, Ar-C*H*], 3.79 [s, 3H, OC*H*$_3$] 2.03 [s, 3H, C≡C*H*$_3$]. **^{13}C{^{1}H}-NMR** (100.7 MHz, $CDCl_3$, 300 K): δ [ppm] = 159.1 [s, Ar-*C*], 132.9 [s, Ar-*C*H], 116.3 [s, Ar-*C*], 114.0 [s, Ar-*C*H], 84.2 [s, *C*≡CCH$_3$], 79.6 [s, C≡*C*CH$_3$], 55.4 [s, O*C*H$_3$], 4.40 [s, C≡*C*H$_3$].

NMR Spectra

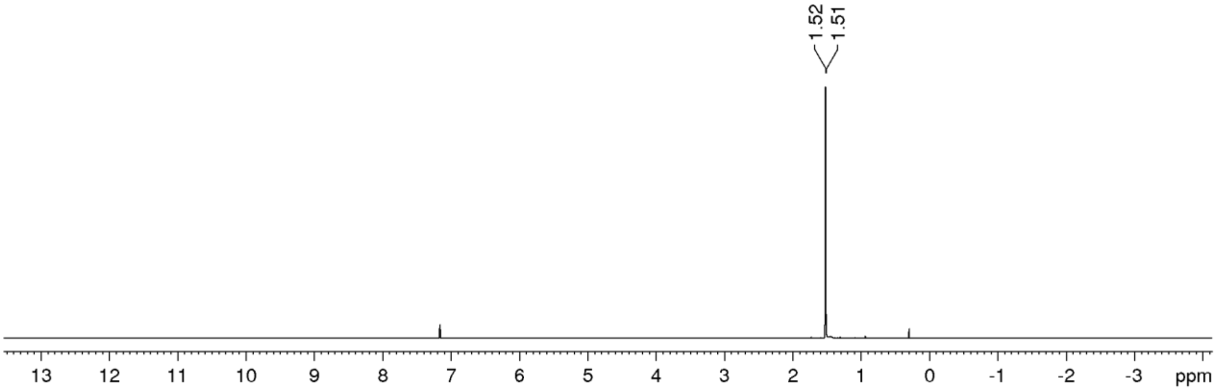

Figure S1: ^{1}H NMR (C_6D_6, 300.3 MHz, 300 K) of **W2F3**.

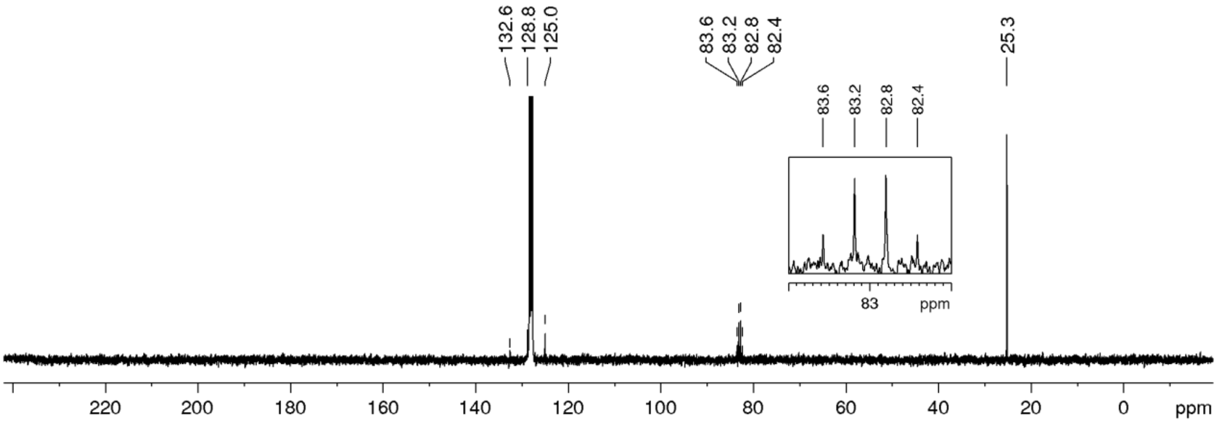

Figure S2: ^{13}C NMR (C_6D_6, 75.5 MHz, 300 K) of **W2F3**.

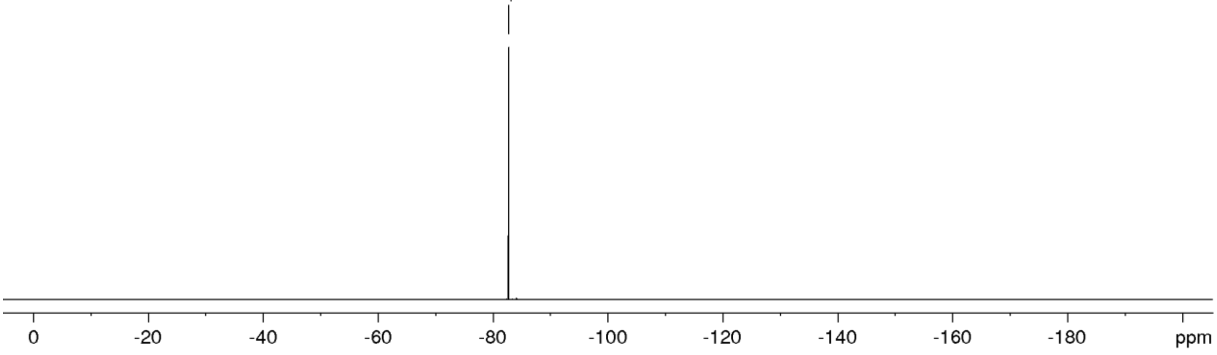

Figure S3: ^{19}F NMR (C_6D_6, 282.5 MHz, 300 K) of **W2F3**.

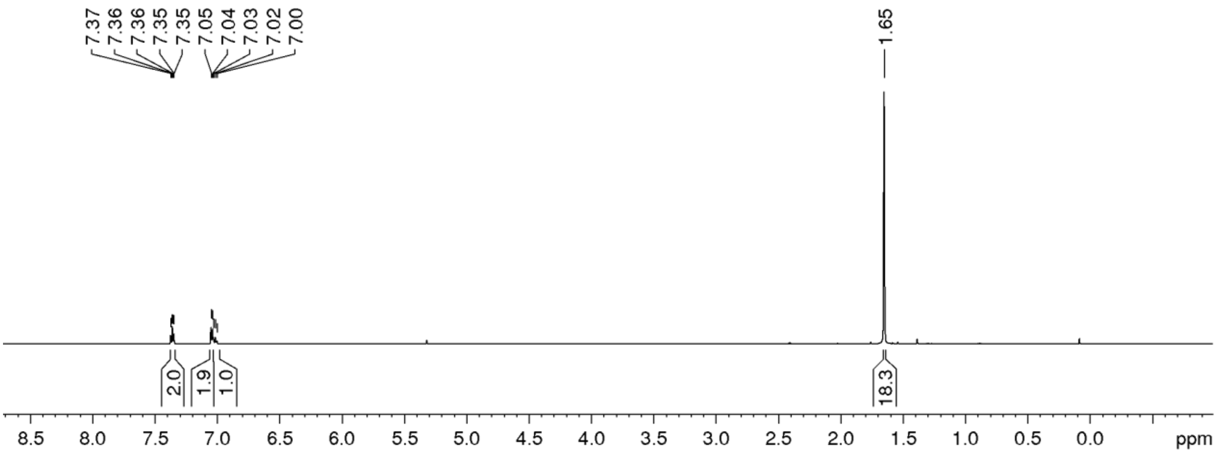

Figure S4: ^{1}H NMR (CD_2Cl_2, 600.1 MHz, 300 K) of **W^{Ph}F3**.

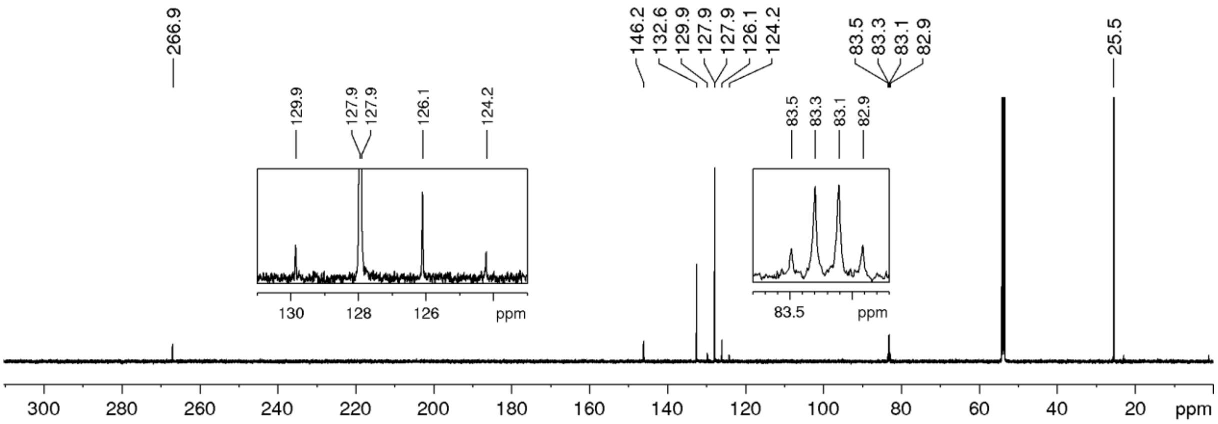

Figure S5: ^{13}C NMR (CD_2Cl_2, 150.9 MHz, 300 K) of **W^{Ph}F3**.

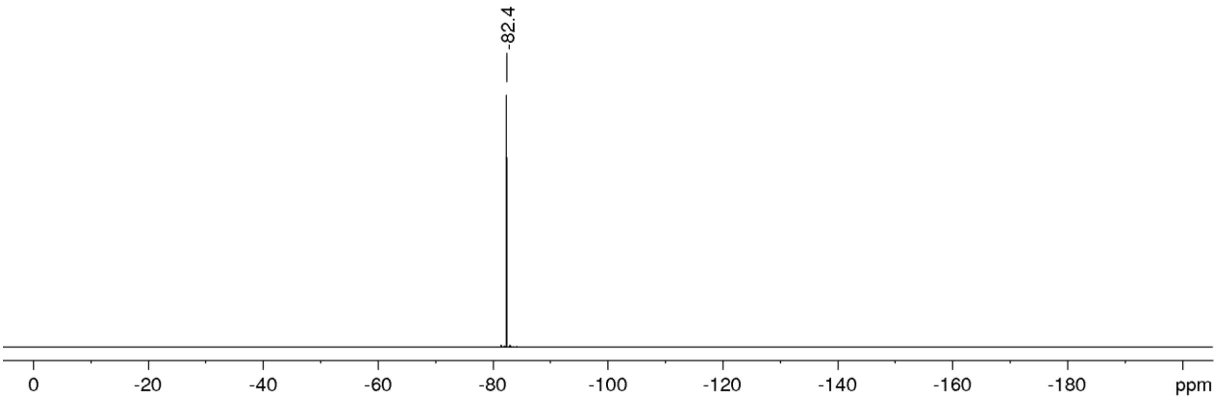

Figure S6: ^{19}F NMR (CD_2Cl_2, 282.5 MHz, 300 K) of **W^{Ph}F3**.

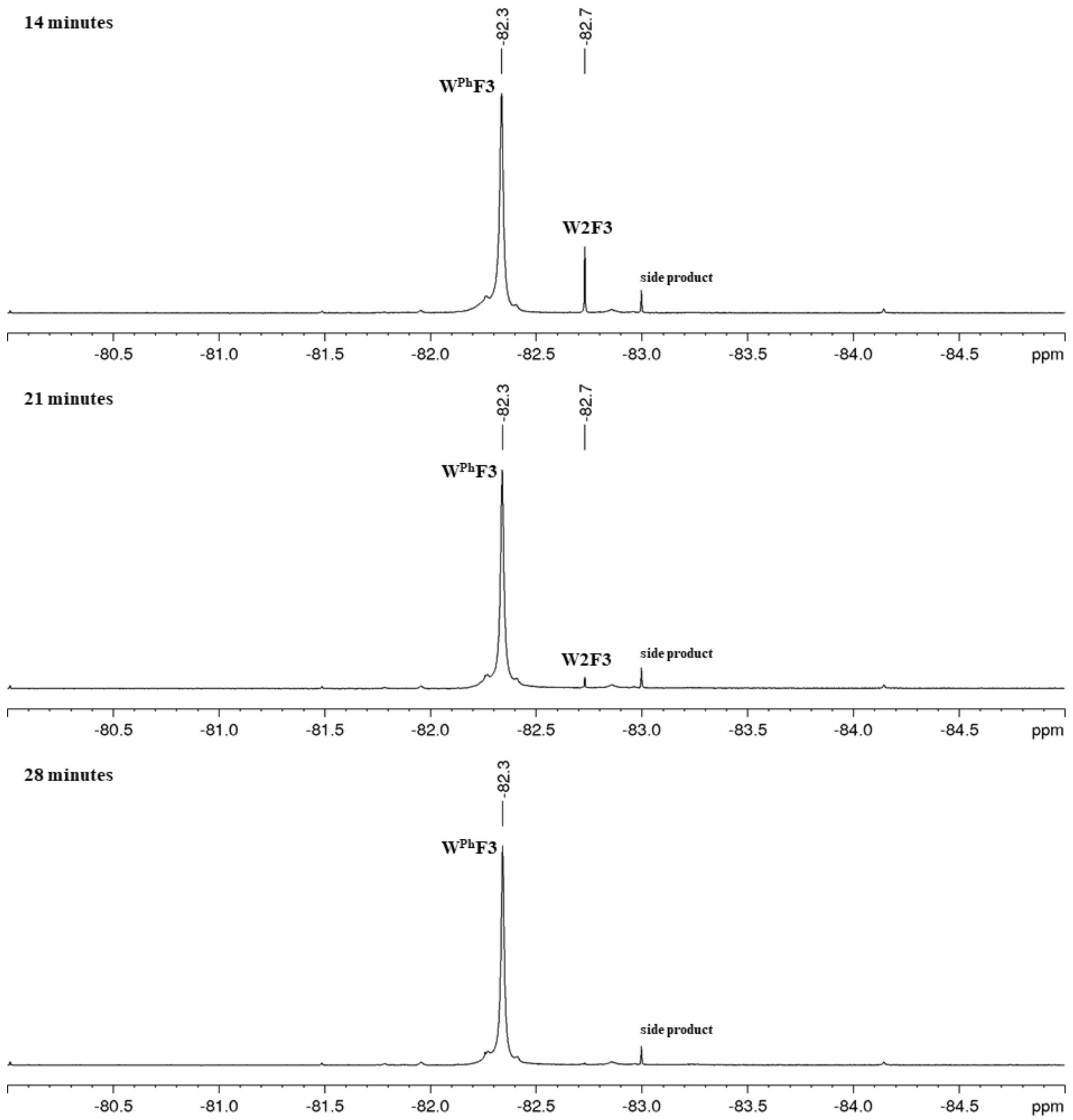

Figure S7: ^{19}F NMR (C_6D_6, 282.5 MHz, 300 K) of the stoichiometric reaction of **W2F3** (5.0 mg, 4.4 µmol) with two equivalents of 1-phenyl-1-propyne (1.0 mg, 8.8 µmol) to **W^{Ph}F3**.

Catalytic studies

Comparison of catalytic activities. Under an argon atmosphere, a flask was charged with the substrate (0.25 mmol), molecular sieves 5 Å (250 mg), 0.05 mL *n*-decane and toluene (internal alkynes: 1.25 mL, terminal alkynes: 12 mL). Then the catalyst (0.5 mol % **W2F3**, 1 mol % **Mo2F6**, 1 mol % **W^{Ph}F3**) was added and samples (internal alkynes: 0.05 mL, terminal alkynes: 0.25 mL) were taken for gas chromatographic analysis in certain intervals. The samples were filtered through a small patch of alumina and washed with diethyl ether.

General procedure for self-metathesis. Under an argon atmosphere, a flask was charged with the substrate (0.5 mmol), molecular sieves 5 Å (500 mg) and toluene (internal alkynes: 2.5 mL, terminal alkynes: 24 mL). Then the catalyst (0.5 mol % **W2F3**, 1 mol % **W^{Ph}F3**) was added and the mixture was stirred for 2 h at room temperature. The catalyst and the molecular sieves were removed by filtration through alumina and the solvent was evaporated. The crude reaction product was purified by flash chromatography on silica gel with ethyl acetate-hexane (1:8).

General procedure for RCAM. Under an argon atmosphere, a flask was charged with the substrate (0.5 mmol), molecular sieves 5 Å (1.0 g) and toluene (24 mL). Then the catalyst (1 mol % **W2F3**, 2 mol % **W^{Ph}F3**) was added and the mixture was stirred for 2 h at room temperature. The catalyst and the molecular sieves were removed by filtration through alumina and the solvent was evaporated. The crude reaction product was purified by flash chromatography on silica gel with ethyl acetate-hexane (1:8).

General procedure for ACM. Under an argon atmosphere, a flask was charged with the substrate (0.5 mmol), TMS-propyne or TMS-acetylene (1.0 mmol), molecular sieves 5 Å (500 mg) and toluene (internal alkynes: 2.5 mL, terminal alkynes: 24 mL). Then the catalyst (1 mol % or 2 mol % **W2F3**) was added and the mixture was stirred for 2 h at room temperature. The catalyst and the molecular sieves were removed by filtration through alumina and the solvent was evaporated. The crude reaction product was purified by flash chromatography on silica gel with ethyl acetate-hexane (1:10).

Catalysis products

1,6-Bis(benzyloxy)hex-3-yne.[4] colorless oil; **^{1}H-NMR** (200.1 MHz, $CDCl_3$, 300 K): δ [ppm] = 7.27–7.16 [m, 10H, Ar-C*H*], 4.46 [s, 4H, OCH_2], 3.48 [t, 4H, ^3J = 7.0 Hz, OCH_2], 2.40 [t, 4H, ^3J = 7.0 Hz, CH_2]. **^{13}C{^{1}H}-NMR** (75.5 MHz, $CDCl_3$, 300 K): δ [ppm] = 138.2 [s, Ar-*C*], 128.3 [s, Ar-*C*H], 127.6 [s, Ar-*C*H], 127.5 [s, Ar-*C*H], 77.9 [s, *C*≡*C*], 72.9 [s, O*C*H_2], 68.7 [s, O*C*H_2], 20.2 [s, *C*H_2].

1,6-Bis(4-methoxybenzoyloxy)hex-3-yne.[4] colorless solid; **^{1}H-NMR** (300.1 MHz, $CDCl_3$, 300 K): δ [ppm] = 7.99 [m, 4H, Ar-C*H*], 6.89 [m, 4H, Ar-C*H*], 4.35 [t, 4H, ^3J = 6.9 Hz, CH_2], 3.85 [s, 6H, OCH_3], 2.62 [t, 4H, ^3J = 6.9 Hz, CH_2]. **^{13}C{^{1}H}-NMR** (75.5 MHz, $CDCl_3$, 300 K): δ [ppm] = 166.2 [s, *C*=O], 163.5 [s, Ar-*C*], 131.8 [s, Ar-*C*H], 122.6 [s, Ar-*C*], 113.7 [s, Ar-*C*H], 76.9 [s, *C*≡*C*] 62.9 [s, *C*H_2], 55.6 [s, O*C*H_3], 19.5 [s, *C*H_2].

***meta*-Xylol-cyclophane.**[5] colorless solid; **^{1}H-NMR** (300.1 MHz, $CDCl_3$, 300 K): δ [ppm] = 8.30 [m, 1H, Ar-C*H*], 7.21−7.25 [m, 1H, Ar-C*H*], 6.70−7.03 [m, 1H, Ar-C*H*], 4.46 [s, 4H, OCH_2], 3.63−6.67 [m, 4H, CH_2], 2.44−2.47 [m, 2H, C≡CCH_2]. **^{13}C{^{1}H}-NMR** (75.5 MHz, $CDCl_3$, 300 K): δ [ppm] = 139.8 [s, Ar-*C*], 127.7 [s, Ar-*C*H], 125.1 [s, Ar-*C*H], 124.5 [s, Ar-*C*H], 79.6 [s, *C*≡*C*], 72.2 [s, O*C*H_2], 69.0 [s, O*C*H_2], 21.0 [s, C*C*H_2].

***ortho*-[10]Cyclophane.**[5] colorless solid; **^{1}H-NMR** (300.1 MHz, $CDCl_3$, 300 K): δ [ppm] = 7.67−7.73 [m, 2H, Ar-C*H*], 7.51−7.60 [m, 2H, Ar-C*H*], 4.46 [t, 4H, ^{3}J = 5.6 Hz, OCH_2], 2.53–2.57 [m, 4H, ^{3}J = 5.7 Hz, CH_2]. **^{13}C{^{1}H}-NMR** (75.5 MHz, $CDCl_3$, 300 K): δ [ppm] = 167.9 [s, *C*=O], 133.2 [s, Ar-*C*], 131.0 [s, Ar-*C*H], 128.5 [s, Ar-*C*H], 79.0 [s, *C*≡*C*], 62.8 [s, OCH_2], 19.8 [s, CH_2].

4-(Benzyloxy)but-1-yn-1-yl)trimethylsilane.[8] colorless liquid; **^{1}H-NMR** (200.1 MHz, $CDCl_3$, 300 K): δ [ppm] = 7.29−7.37 [m, 5H, Ar-C*H*], 4.57 [s, 2H, OCH_2], 3.61 [t, 2H, ^{3}J = 7.1 Hz, OCH_2], 2.56 [t, 2H, ^{3}J = 7.1 Hz, CH_2], 0.16 [s, 9H, Si$(CH_3)_3$]. **^{13}C{^{1}H}-NMR** (75.5 MHz, $CDCl_3$, 300 K): δ [ppm] = 138.3 [s, Ar-*C*], 128.5 [s, Ar-*C*H], 127.8 [s, Ar-*C*H], 103.9 [s, C≡*C*Si$(CH_3)_3$], 85.9 [s, *C*≡CSi$(CH_3)_3$], 73.1 [s, ArCH_2O], 68.5 [s, CH_2], 21.5 [s, CH_2], 0.2 [s, Si$(CH_3)_3$].

Trimethyl(phenylethynyl)silane.[9] colorless oil; **^{1}H-NMR** (300.1 MHz, $CDCl_3$, 300 K): δ [ppm] = 7.27−7.33 [m, 3H, Ar-C*H*], 7.45−7.48 [m, 2H, Ar-C*H*], 0.26 [s, 9H, Si$(CH_3)_3$]. **^{13}C{^{1}H}-NMR** (75.5 MHz, $CDCl_3$, 300 K): δ [ppm] = 132.1 [s, Ar-*C*H], 128.6 [s, Ar-*C*H], 128.3 [s, Ar-*C*H], 123.2 [s, Ar-*C*], 105.2 [s, *C*≡CSi$(CH_3)_3$], 94.2 [s, C≡*C*Si$(CH_3)_3$], 0.1 [s, Si$(CH_3)_3$].

Tolane.[10] colorless solid; **^{1}H-NMR** (300.1 MHz, $CDCl_3$, 300 K): δ [ppm] = 7.52–7.57 [m, 4H, Ar-C*H*], 7.33–7.39 [m, 6H, Ar-C*H*]. **^{13}C{^{1}H}-NMR** (75.5 MHz, $CDCl_3$, 300 K): δ [ppm] = 131.8 [s, Ar-*C*H], 128.5 [s, Ar-*C*H], 128.4 [s, Ar-*C*H], 123.4 [s, Ar-*C*], 89.5 [s, *C*≡*C*].

1,2-Bis-4-tolylethyne.[11] colorless solid; **^{1}H-NMR** (300.1 MHz, $CDCl_3$, 300 K): δ [ppm] = 7.40–7.44 [m, 4H, Ar-C*H*], 7.13–7.16 [m, 4H, Ar-C*H*], 2.37 [s, 6H, PhCH_3]. **^{13}C{^{1}H}-NMR** (75.5 MHz, $CDCl_3$, 300 K): δ [ppm] = 138.3 [s, Ar-*C*], 131.6 [s, Ar-*C*H], 129.2 [s, Ar-*C*H], 120.5 [s, Ar-*C*], 89.0 [s, *C*≡*C*], 21.6 [s, CH_3].

1,2-Bis(4-bromophenyl)ethyne.[12] colorless solid; **^{1}H-NMR** (400.4 MHz, $CDCl_3$, 300 K): δ [ppm] = 7.47–7.50 [m, 4H, Ar-C*H*], 7.36–7.39 [m, 4H, Ar-C*H*]. **^{13}C{^{1}H}-NMR** (100.7 MHz, $CDCl_3$, 300 K): δ [ppm] = 133.1 [s, Ar-*C*H], 131.8 [s, Ar-*C*H], 122.9 [s, Ar-*C*], 122.0 [s, Ar-*C*], 89.6 [s, *C*≡*C*].

1,2-Bis(4-methoxyphenyl)ethyne.[10] colorless solid; **^{1}H-NMR** (400.4 MHz, $CDCl_3$, 300 K): δ [ppm] = 7.43–7.47 [m, 4H, Ar-C*H*], 6.85–6.89 [m, 4H, Ar-C*H*], 3.82 [s, 6H, OCH_3]. **^{13}C{^{1}H}-NMR** (100.7 MHz, $CDCl_3$, 300 K): δ [ppm] = 159.3 [s, Ar-*C*], 133.0 [s, Ar-*C*H], 115.9 [s, Ar-*C*], 114.1 [s, Ar-*C*H], 88.1 [s, *C*≡*C*], 55.4 [s, OCH_3].

Conversion versus time diagrams

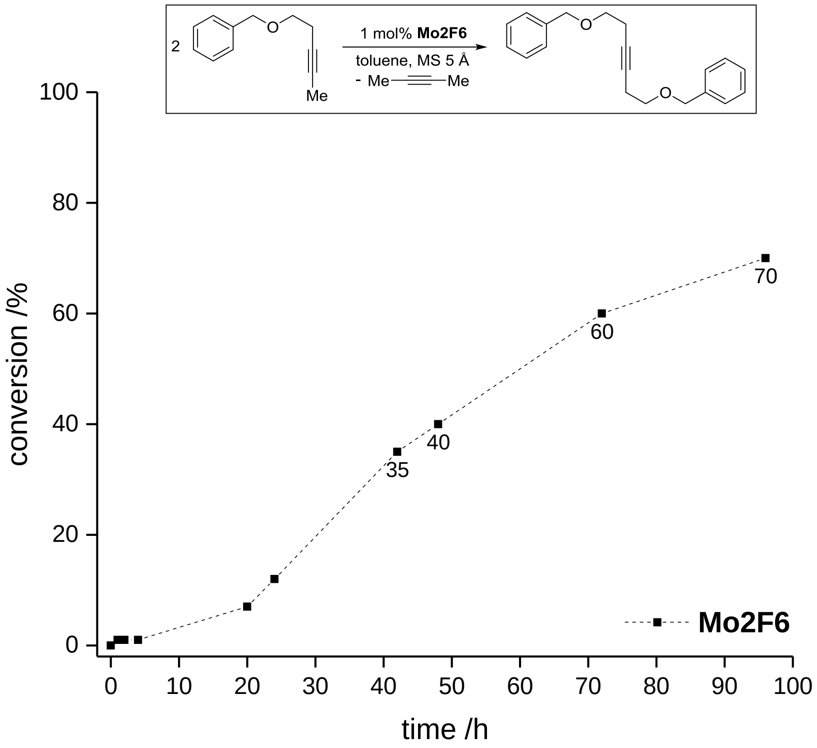

Figure S8: Conversion versus time diagram for self-metathesis of 3-pentynyl benzyl ether with 1 mol % **Mo2F6**.

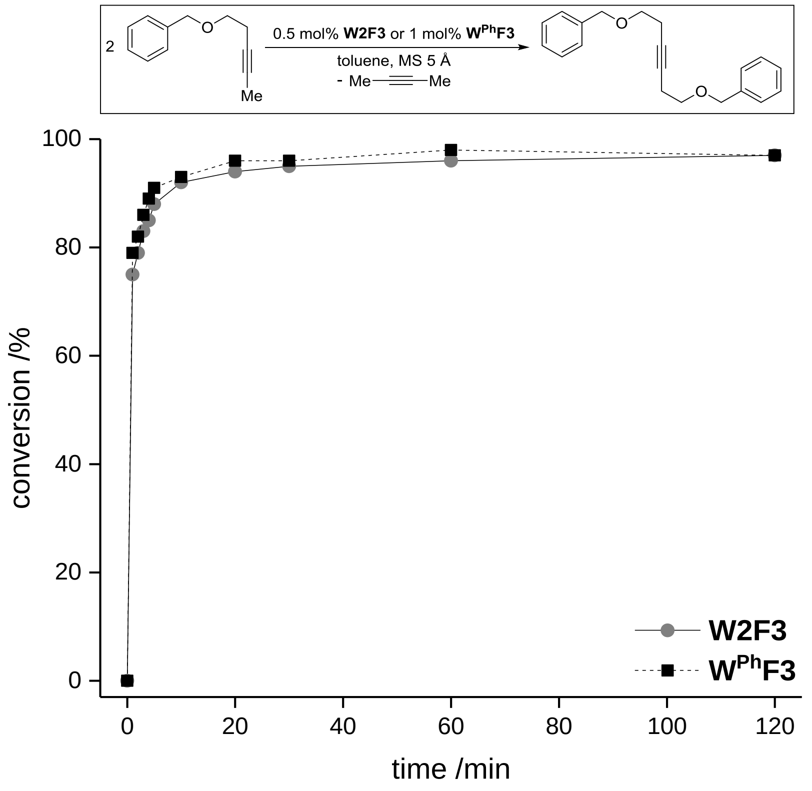

Figure S9: Conversion versus time diagram for self-metathesis of 3-pentynyl benzyl ether with 0.5 mol % **W2F3** or 1 mol % **W^{Ph}F3**.

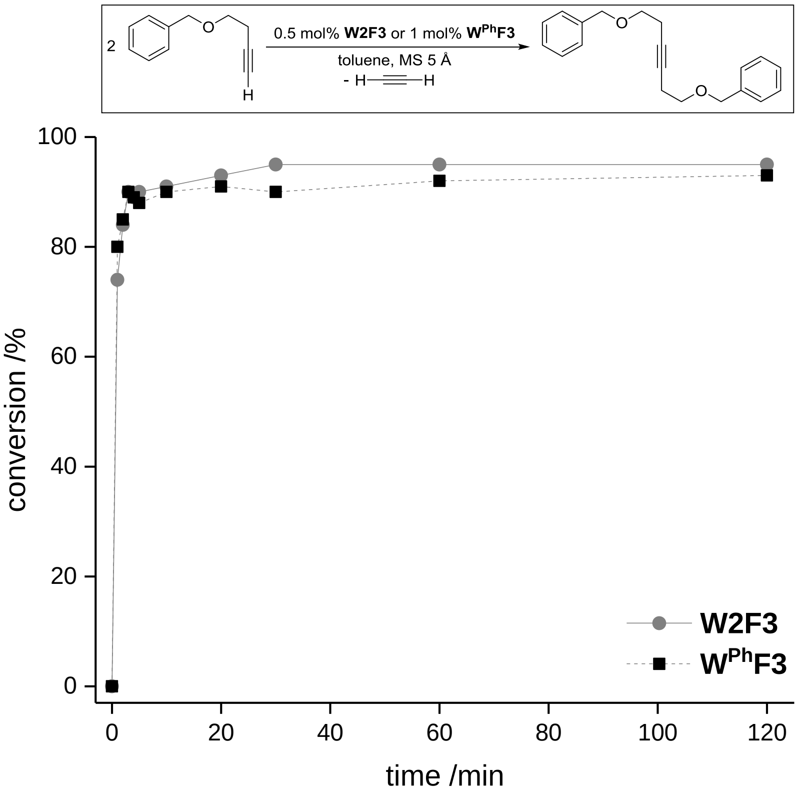

Figure S10: Conversion versus time diagram for self-metathesis of 3-butynyl benzyl ether with 0.5 mol % **W2F3** or 1 mol % **W^{Ph}F3**.

X-ray structure analysis

Numerical data are collected in table S1. Single crystals were mounted on glass fibers (**W2F3**·$NHMe_2$ and **W^{Ph}F3**) or on top of a human hair (**W2F3**) in perfluorinated inert oil. Measurements were performed on Oxford Diffraction Xcalibur diffractometers using monochromated Mo Kα radiation. Absorption corrections were applied on the basis of multi-scans and additional absorption correction based on face indexing and integration on a Gaussian grid. Data reduction was performed with CrysalisPro.[13] The structures were solved by intrinsic phasing with SHELXT-2014/5[14] and refined on F^2 using the program SHELXL-2017/1[15]. The Hydrogen H1 on the nitrogen atom in **W2F3**·$NHMe_2$ was refined freely. All other H atoms in all the reported crystal structures were placed in idealized positions and refined using a riding model.

W2F3. Measurement and refinement details: Crystals were mounted on the diffractometer on top of a human hair with inert oil at 250 K. The crystal was then slowly cooled down on the diffractometer to 100 K in the nitrogen gas stream before the data collection was started. The asymmetric unit consists of two halves of two independent molecules. The tungsten atoms are disordered over four positions each resulting in eight positions because of the inversion centre on the tungsten-tungsten bonds. On

both molecules one alkoxide ligand moiety is refined as disordered over two positions each (Occupancies: O3 / O3A: 48.7 % / 51.3 %; O6 / O6A: 63.5 % / 36.5 %). Occupancies as well as bond distances for each tungsten atom are given in Table S1.

Table S1: Occupancies and bond distances for the tungsten atoms.

atom	occupancy	$d_{(W\text{-}W)}$ [Å]	$d_{(W\text{-}O1\|4)}$ [Å]	$d_{(W\text{-}O2\|5)}$ [Å]	$d_{(W\text{-}O3\|O6/O3A\|O6A)}$ [Å]
W1	85.7(2) %	2.3298(7)	1.920(5)	1.879(7)	1.832(11)/1.927(14)
W1A	3.1(2) %	2.293(14)	1.993(9)	2.081(10)	2.021(13)/2.154(16)
W1B	8.6(2) %	2.317(10)	2.043(7)	2.277(7)	2.095(11)/1.908(14)
W1C	2.6(1) %	2.31(2)	1.975(12)	2.128(12)	2.289(15)/2.169(17)
W2	63.4(2) %	2.3294(8)	1.919(7)	1.876(8)	1.888(9)/2.46(3)
W2A	23.6(1) %	2.325(3)	1.856(7)	1.899(7)	2.577(9)/1.87(3)
W2B	2.5(1) %	2.385(19)	2.055(11)	2.075(11)	2.211(13)/2.07(3)
W2C	10.5(1) %	2.315(6)	1.925(7)	2.245(8)	2.035(10)/1.92(4)

This structure only confirms the connectivity of this molecule and discussion of any bond length is not meaningful. This kind of disorder is an inherent problem in M_2X_6 systems resulting from a pseudo octahedral ligand arrangement and an internal flip mechanism for the metal atoms.[16] The different positions of the metal atoms on the disordered metal-metal vector occupy the edges of an imaginary cuboid.

W^{Ph}F3. Two alkoxide ligands were refined with a disorder model comprising two or three different positions, respectively. The simulated precession images of this dataset (see Figures S11 and S12) show very weak satellite peaks in the 0kl and hk0 planes for low diffraction angles only. Meaningful integration of these peaks was not possible. We assume that these satellite peaks result from a modulation of the crystal structure and that the refined disorder model is the most eligible approximation.

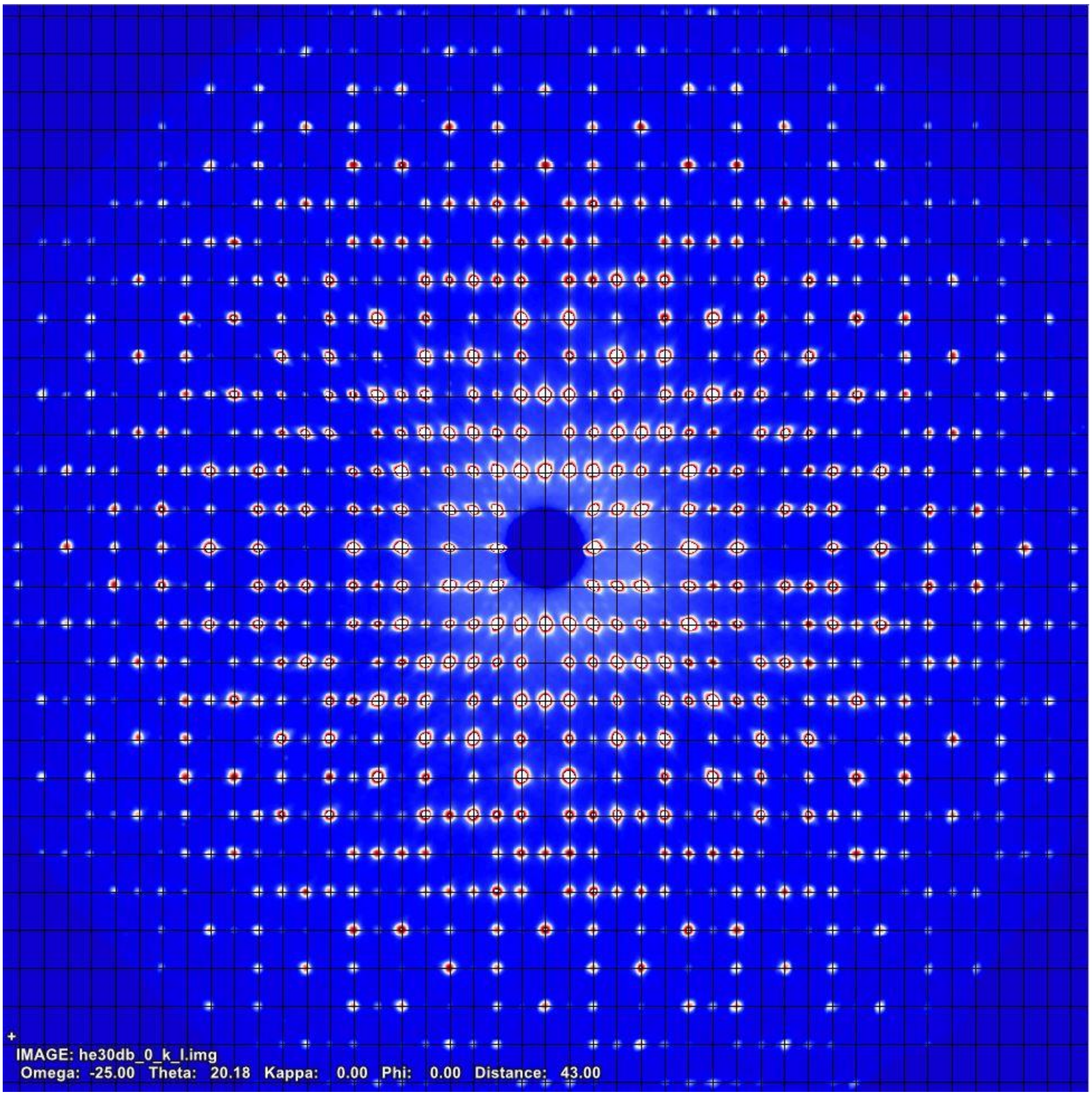

Figure S11: Simulated precession image of the 0kl plane showing weak satellite peaks for small diffraction angles.[13]

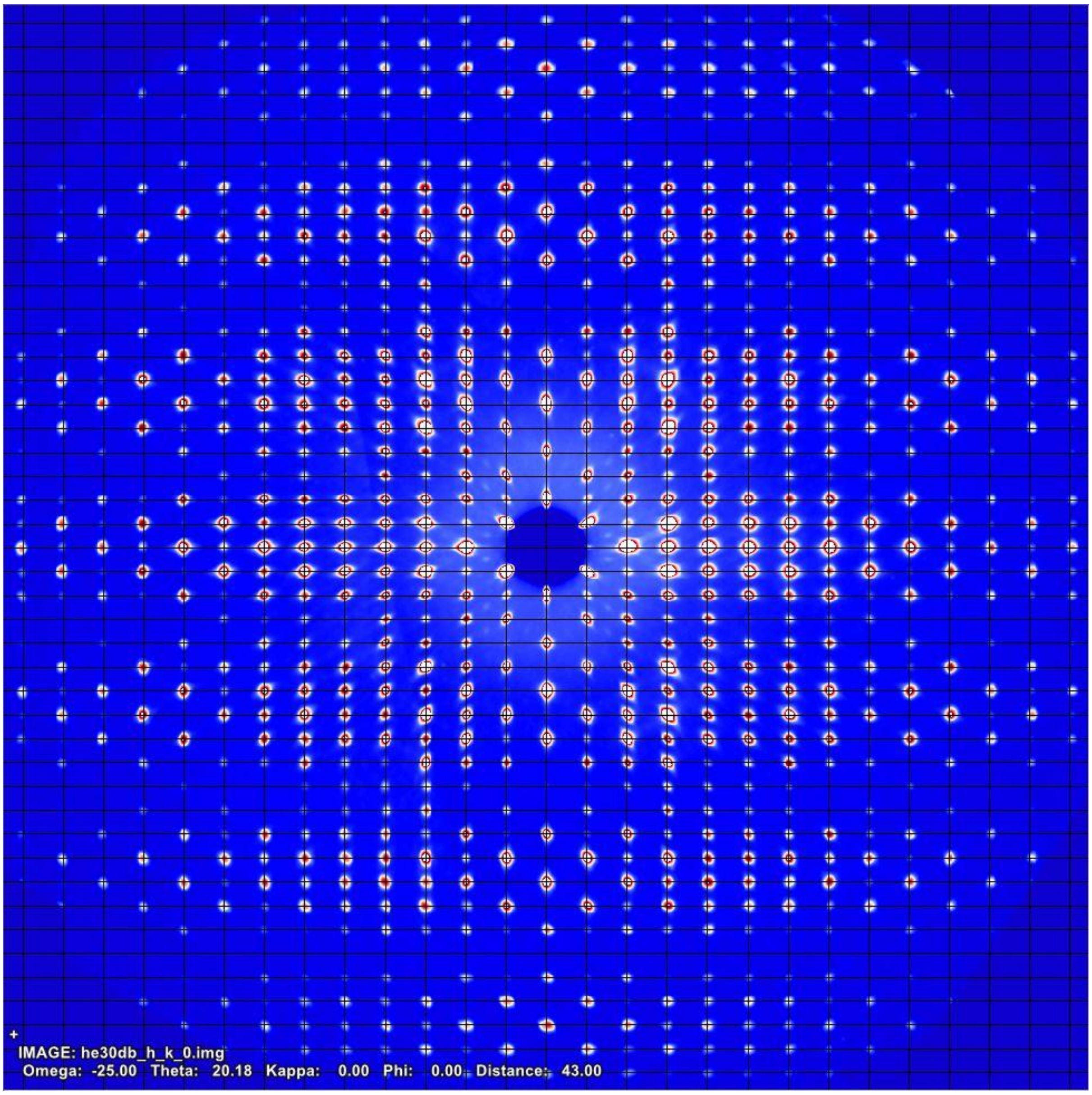

Figure S12: Simulated precession image of the hk0 plane showing weak satellite peaks for small diffraction angles.[13]

Complete data have been deposited with the Cambridge Crystallographic Data Centre under the CCDC numbers 1850924−1850926. These data can be obtained free of charge from http://www.ccdc.cam.ac.uk/.

Table S2: Selected crystallographic data for compounds measured by X-ray structure analysis.

	**W2F3**·$NHMe_2$	**W2F3**	**W^{Ph}F3**
CCDC	1850924	1850925	1850926
Formula	$C_{26}H_{43}F_{18}NO_6W_2$	$C_{24}H_{36}F_{18}O_6W_2$	$C_{19}H_{23}F_9O_3W$
M [g mol^{-1}]	1175.31	1130.23	654.22
T/K	100(2)	100(2)	100(2)
λ/Å	0.71073	0.71073	0.71073
Crystal system	triclinic	monoclinic	monoclinic
Space group	$P\bar{1}$	$P2_1/c$	$P2_1/c$
a/Å	10.6380(4)	18.3989(8)	11.2694(2)
b/Å	10.7814(3)	17.5157(6)	18.1575(4)
c/Å	17.1943(6)	11.0958(6)	11.9490(3)
α/°	91.326(3)	90	90
β/°	92.636(3)	97.361(4)	107.613(2)
γ/°	108.043(3)	90	90
V/Å^3	1871.64(11)	3546.4(3)	2330.43(9)
Z	2	4	4
D_x/g cm^{-3}	2.085	2.117	1.865
μ/mm^{-1}	6.269	6.612	5.045
F(000)	1128	2152	1264
Cryst. habitus	black-brown dichroic prism	red needle	brown, irregular shape
Cryst. size/mm	0.144 x 0.119 x 0.071	0.397 x 0.088 x 0.085	0.328 x 0.258 x 0.194
Theta range/°	2.275 to 28.700°	2.232 to 29.574	2.243–29.129
Reflections collected	96247	120724	106472
Indep. reflections	9663	9947	6261
R(int)	0.0682	0.0784	0.0297
Data / restraints / parameters	9663 / 0 / 496	9947 / 56 / 485	6261 / 61 / 388
GooF	1.041	1.021	1.055
R1 (F, >4σ(F))	0.0259	0.0567	0.0151
wR2 (F2, all refl.)	0.0554	0.1634	0.0354
max. Δρ/e Å^{-3}	2.174 and -0.840	4.176 and -1.937	0.728 /–0.452

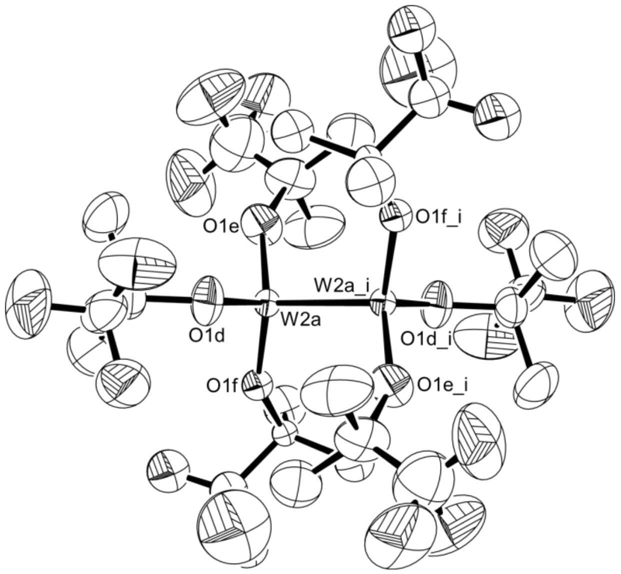

Figure S13: ORTEP diagram of **W2F3**. Hydrogen atoms and disordered tungsten atoms and alkoxides are omitted for clarity.

References

[1] T. M. Gilbert, A. M. Landes, R. D. Rogers, *Inorg. Chem.* **1992**, *31*, 3438–3444.

[2] M. H. Chisholm, F. A. Cotton, M. Extine, M. Millar, B. Ray Stults, *J. Am. Chem. Soc.* **1976**, *98*, 4486–4491.

[3] J. A. Samuels, K. Folting, J. C. Huffman, K. G. Caulton, *Chem. Mater.* **1995**, *7*, 929–935.

[4] B. Haberlag, X. Wu, K. Brandhorst, J. Grunenberg, C. G. Daniliuc, P. G. Jones, M. Tamm, *Chem. Eur. J.* **2010**, 16, 8868−8877.

[5] B. Haberlag, M. Freytag, C. G. Daniliuc, P. G. Jones, M. Tamm, *Angew. Chem. Int. Ed.* **2012**, *51*, 13019–13022.

[6] S. Beer, K. Brandhorst, J. Grunenberg, C. G. Hrib, P.G. Jones, M. Tamm, *Org. Lett.* **2008**, *10*, 981−984.

[7] T. Fujihara, Y. Tani, K. Semba, J. Terao, Y. Tsuji, *Angew. Chem. Int. Ed.* **2012**, *51*, 11487–11490.

[8] D. P. Estes, C. Bittner, Ò. Àrias, M. Casey, A. Fedorov, M. Tamm, C. Copéret, *Angew Chem. Int. Ed.* **2016**, *55*,13960 –13964.

[9] P. C. Bulman Page, S. Rosenthal, *Tetrahedron* **1990**, *46*, 2573–2586.

[10] Z. Novák, P. Nemes, A. Kotschy, *Org. Lett.* **2004**, *6*, 4917–4920.

[11] J. Moon, M. Jeong, H. Nam, J. Ju, J. H. Moon, H. M. Jung, S. Lee, *Org. Lett.* **2008,** *10*, 945–948.

[12] E.-K- Yum, J.-W. Son, S.-K. Kim, S.-N. Kim, K.-M. Kim, C.-W. Lee, *Bull. Korean Chem. Soc.* 2**010,** *31*, 2097–2099.

[13] Rigaku Oxford Diffraction, *CrysAlisPRO Software System*, versions 1.171.38.43 (**2015**) and 1.171.39.46 (**2018**), Rigaku Corporation, Oxford, UK.

[14] G. M. Sheldrick, *Acta Cryst. Sect. A: Found. Adv.* **2015**, A71, 3–8.

[15] G. M. Sheldrick, *Acta Cryst. Sect. C: Struct. Chem.* **2015**, C71, 3–8.

[16] a) R. H. Cayton, M. H. Chisholm, *Inorg. Chem.* **1991**, *30*, 1422–1425; b) M. H. Chisholm, C. M. Cook, J. C. Huffman, W. E. Streib, *J. Chem. Soc. Dalton Trans* **1991**, 929–937; c) T. M. Gilbert, A. M. Landes, R. D. Rogers, *Inorg. Chem.* **1992**, *31*, 3438–3444; d) T. M. Gilbert, C. B. Bauer,

A. H. Bond, R. D. Rogers, *Polyhedron* **1999**, *18*, 1293–1301; e) K. L. Fujdala, T. D. Tilley, *Chem. Mater.* **2004**, *16*, 1035–1047; f) T. M. Gilbert, J. C. Littrell, C. E. Talley, M. A. Vance, R. F. Dallinger, R. D. Rogers, *Inorg. Chem.* **2004**, *43*, 1762–1769; g) M. H. Chisholm, J. C. Gallucci, C. B. Hollandsworth, *Polyhedron* **2006**, *25*, 827–833; h) C. Bittner, H. Ehrhorn, D. Bockfeld, K. Brandhorst, M. Tamm, *Organometallics* **2017**, 36, 3398–3406.
